# Structured Care and Self-Management Education for Persons with Parkinson’s Disease: Why the First Does Not Go without the Second—Systematic Review, Experiences and Implementation Concepts from Sweden and Germany

**DOI:** 10.3390/jcm9092787

**Published:** 2020-08-28

**Authors:** Jenny Tennigkeit, Tim Feige, Maria Haak, Carina Hellqvist, Ümran S. Seven, Elke Kalbe, Jaqueline Schwarz, Tobias Warnecke, Lars Tönges, Carsten Eggers, Kai F. Loewenbrück

**Affiliations:** 1 Department of Neurology, University Hospital Dresden, 01307 Dresden, Germany; jenny.tennigkeit@uniklinikum-dresden.de (J.T.); tim.feige@uniklinikum-dresden.de (T.F.); 2 German Center for Neurodegenerative Diseases (DZNE) Dresden, 01307 Dresden, Germany; 3 Faculty of Health Sciences, Kristianstad University, 291 88 Kristianstad, Sweden; Maria.haak@hkr.se; 4 Department of Health Sciences, Faculty of Medicine, Lund University, 221 00 Lund, Sweden; 5 Department of Neurology, University Hospital Linköping, 58185 Linköping, Sweden; Carina.hellqvist@regionostergotland.se; 6 Medical Psychology, Neuropsychology and Gender Studies and Center for Neuropsychological Diagnostics and Intervention (CeNDI), Faculty of Medicine and University Hospital Cologne, University of Cologne, 50937 Cologne, Germany; Uemran.seven@uk-koeln.de (Ü.S.S.); elke.kalbe@uk-koeln.de (E.K.); 7 Tumaini Institut für Präventionsmanagement GmbH, 01217 Dresden, Germany; jschwarz@tumaini.de; 8 Department of Neurology, University Hospital Münster, 48149 Münster, Germany; Tobias.Warnecke@ukmuenster.de; 9 Department of Neurology, St. Josef-Hospital, Ruhr-University Bochum, 44801 Bochum, Germany; Lars.toenges@rub.de; 10 Neurodegeneration Research, Centre for Protein Diagnostics (ProDi), Ruhr University, 44801 Bochum, Germany; 11 Department of Neurology, University Hospital Marburg, 35033 Marburg, Germany; Carsten.eggers@uk-gm.de

**Keywords:** networks, Parkinson disease, integrated care, self-management, education

## Abstract

Integrated care is regarded as a key for care delivery to persons with chronic long-term conditions such as Parkinson’s disease. For persons with Parkinson’s disease, obtaining self-management support is a top priority in the context of integrated care. Self-management is regarded as a crucial competence in chronic diseases since the affected persons and their caregivers inevitably take up the main responsibility when it comes to day-to-day management. Formal self-management education programs with the focus on behavioral skills relevant to the induction and maintenance of behavioral change have been implemented as a standard in many chronic long-term conditions. However, besides the example of the Swedish National Parkinson School, the offers for persons with Parkinson’s disease remain fragmented and limited in availability. Today, no such program is implemented as a nationwide standard in Germany. This paper provides (1) a systematic review on structured self-management education programs specifically designed or adopted for persons with Parkinson’s disease, (2) presents the Swedish National Parkinson School as an example for a successfully implemented nationwide program and (3) presents a concept for the design, evaluation and long-term implementation of a future-orientated self-management education program for persons with Parkinson’s disease in Germany.

## 1. Introduction

### 1.1. Integrated Care Concepts and Self-Management

Integrated care concepts (ICCs) are a core strategy to meet the health care challenge of age-related chronic degenerative diseases like Parkinson’s disease (PD) [1]. Common definitions describe integrated care as a person-centered approach delivering comprehensive coordinated care involving a multi- or interdisciplinary team across settings and levels of care [2]. Persons with chronic conditions (PwCD) are inevitably involved in their own care: They “cannot not manage” their diseases [3]. No matter how comprehensive an ICC might be, health care professionals are only intermittently involved and cannot alleviate the majority of disease sequelae. PwCDs and their social surroundings are inevitably most important when it comes to day-to-day management [3]. Thus, PwCDs should be considered as members of their own interdisciplinary team of healthcare providers.

PD is a prime example of a disease that confronts affected persons with high and evolving challenges in taking up this decade-long task: persons with Parkinson’s disease (PwPDs) experience an increasingly complex disease burden with an array of motor and non-motor symptoms, require a multidimensional pharmaceutical and non-pharmaceutical treatment regimen and experience a varying therapeutic effectiveness along the disease course.

Self-management is defined as “tasks that individuals must undertake to live with one or more chronic conditions. These tasks include having the confidence to deal with (1) medical management, (2) role management and (3) emotional management of their conditions” [4]. A recent expert panel on ICCs for PwPDs recommended self-management support as one of 30 components [5]. In contrast, PwPDs even give self-management support a top priority when asked about their requirements for ICCs: in two independent studies on the needs of PwPDs in ICCs, self-management support evolved as the top requirement [6,7]. Since ICCs rely to a varying extent on a person’s capacity in self-management, measures to promote self-management should be an inherent part of ICCs [8]. Self-management support measures do not only respect the needs of PwPDs, but also professional health care providers—their capacities to the best care possible rely on a productive participation of PwPDs themselves.

### 1.2. Components of Self-Management and Conceptual Frameworks

Self-management stretches beyond medical management such as taking the prescribed medication and requires the ability to adopt one’s behavior to symptoms or disability and to cope productively with emotions like fear or anxiety. This means that self-management represents a complex cognitive-behavioral challenge, involving the constant adjustment of role-related behaviors and processing of disease-related emotions. Negative emotions such as anxiety are related to lower quality of life (QoL) and higher mortality in PD [9]; this underpins the importance emotional self-management for health-related outcomes in PD.

This explains why unidimensional education initiatives, e.g., solely promoting disease-related knowledge or focusing on isolated behaviors like compliance often failed to be effective [10]. Rather, education programs that incorporate the training of skills required to induce and maintain behavioral changes are required. Only then sustainable improvements in function, emotional state or health-related outcomes can be achieved [3]. A seminal program that puts the training of skills into focus needed for behavioral change is the Chronic Disease Self-Management Program (CDSMP). Skills trained through the program are: (1) problem solving, (2) decision making, (3) resource utilization, (4) forming of a patient/health care provider relationship and (5) taking action [11]. The effectiveness of this program has been illustrated in randomized controlled trials (RCTs) both when implemented as a generic program for persons with various diseases and in disease-specific adaptations [12,13,14]. Improvements in a variety of outcomes could be achieved, such as health-promoting behaviors, function, cognitive symptom management or overall health status.

The program is based on a person-centered conceptual framework related to social cognitive theory [15]. The theory has been extensively validated and claims that for the induction and maintenance of behavioral change both the mastery of the named core skills is necessary, as well as the belief in one’s self-efficacy to perform a certain behavior successfully [16]. An important aspect of self-efficacy is that it can be task-specific and does not necessarily extend to other behaviors [17]. Besides promoting skills mastery in action planning, the following strategies promote behavioral skills and self-efficacy beliefs: symptom reinterpretation—forming of alternative explanations for symptoms to allow for new self-management behaviors; modeling—teaching material reflects the target population and their situation adequately; social persuasion—use of the social context to support health-promoting behaviors, e.g., by group interventions with peers [16,17]. Related to the skill of action planning is self-tailoring. Self-tailoring means the competence to adjust actions or recommended health-promoting behaviors to personal capacities, preferences and living conditions [3]. Self-efficacy-promoting interventions have been found to improve outcomes in several domains, such as HbA1c in diabetes mellitus, various self-management behaviors (e.g., stress coping, pain management, medication adherence), general health status or quality of life [16,17].

### 1.3. Implementation of Self-Management Programs

Even though self-management interventions have been shown to be effective in a variety of settings (e.g., rehabilitation, outpatient or community-based settings) and with varying delivery strategies (e.g., combination with other therapeutic interventions, such as physiotherapy) [8], there is a strong rationale to devote a distinct structured program to self-management education (SME).

The CDSMP is a prime example of such a program, delivered to small groups of PwCDs and care givers in several modules over several weeks. The program can be provided by trained health care providers (train-the-trainer principle) and has been shown to be generalizable to different diseases and cultural settings [16,17]. Such structured group programs do not only reflect the importance of self-management but also facilitate the delivery of self-management support with a standard quality. Moreover, the modular group-based approach facilitates core objectives such as practical training in action planning, modeling or social persuasion due to the regular contact with other PwCDs.

In spite of PD being a prime example of a progressive long-term condition with high demands in self-management competence, access to structured SME programs for PwPDs has lacked behind other comparable diseases. The current work will provide (1) a systematic literature review about structured self-management programs for PwPDs, (2) report the successful nationwide implementation of the National Parkinson School (NPS) in Sweden and (3) present a concept for the design, evaluation and sustainable implementation of a possible future-orientated SME program for PwPDs in Germany.

## 2. Materials and Methods

### Systematic Review

A systematic review was conducted according to Preferred Reporting Items for Systematic Reviews and Meta-Analyses (PRISMA) criteria with the objective to report content, format and outcome of different SME programs specifically designed or adopted for PwPDs [18]. Level of evidence of quantitative studies retrieved was assessed by American Academy for Cerebral Palsy and Developmental Medicine (AACPDM) criteria [19].

Literature research was conducted between May-June 2020 using the databases PubMed and EBSCO (consisting of Cumulative Index to Nursing and Allied Health Literature (CINAHL), American Psychological Association (APA) PsycArticles, APA PsycInfo, Table of Contents (TOC) Premier). No publication date restriction was applied. Search term was: (parkinson*) AND ((“patient education”) OR (“patient school”) OR (“self-management”) OR (“self management”) OR (“selfmanagement”)).

Eligibility criteria for the studies retrieved by the search terms were: (1) inclusion of elements/components specifically dedicated to training of principles/skills/beliefs needed for induction or maintenance of behavioral change (self-management approach); (2) Program either adopted of specifically designed for PwPDs. Exclusion criteria were: (1) posters, books; (2) unidimensional programs: no program elements identifiable specifically dedicated to principles/skills/beliefs needed for behavioral change next to other training elements (e.g., physiotherapy); (3) full text not accessible.

Included studies are presented in separate tables for quantitative and qualitative evaluation studies. Protocols for future planned studies to evaluate SME programs are given in another separate table. For the PRISMA flow diagram checklist, please refer to Figure S1 and Table S1 (Supplementary Materials).

## 3. Results

After removal of duplicates, 631 records were retrieved, of which 551 were excluded after screening of title and abstracts for in-/exclusion criteria. After full-text assessment of the remaining 62 articles, 23 fully met inclusion criteria and were integrated into this systematic review; 20 described the evaluation of self-management interventions at different stages of implementation, and 3 described evaluation protocols of planned studies on future programs. One additional study was included that was not retrieved by the search terms but that otherwise met all inclusion criteria [20].

### 3.1. General Program Description

A total of 18 different programs were described in the 23 publications. All programs were organized in modular small group sessions (2–18 sessions) with peer PwPDs, 8 programs (35%) including caregivers [21,22,23,24,25,26,27,28]. An exception to modular small group sessions was a study that compared 12 weeks of small group sessions (EXCEED) with the same program studied independently at home after one introductory group session [29]. Sessions were scheduled once or twice a week, ranging from 45 to 150 min in duration. Two (11%) programs were restricted to the training of self-management behaviors and associated cognitive behavioral skills [25], 9 programs (50%) combined self-management support with other therapeutic measures, such as physiotherapy (7, 39%) [22,24,30,31,32,33,34], occupational therapy (4, 22%) [21,24,31,32], relaxation and body awareness techniques (7, 39%) [21,22,26,28,30,35,36] or speech therapy (4, 22%) [21,22,32,35]. All programs were carried out by healthcare professionals, several (9, 50%) by a multiprofessional team of up to 8 different healthcare professions [21,23,24,25,28,30,31,32,34]. Whether the executing staff received training in teaching self-management behaviors, related skills and beliefs, was indicated for 5 programs (28%) [26,28,29,32,37]. 2 programs (11%) mentioned the inclusion of trained peer PwPDs into the program delivery teams [26,29]. Most programs did not target specific PD subpopulations, with the exception of the EXCEED program for PwPDs with unipolar major depression [29], and with the exception of the Early Management Program (EMP), the Safe Mobility (SMP) and Falls Prevention Program (FPP), designed to address PwPDs in early, intermediate and advanced diseases stages with shifting focus on different self-management behaviors [22]. For two programs (11%) it was explicitly reported that they were adaptations of generic programs such as the most widely implemented CDSMP [26,29]. Other programs such as the Patient Education Program for PD (PEEP) do not explicitly refer to generic programs like the CDSMP, but still have substantial similarities in objectives, modular organization and program content and thus appear to be inspired by such disease-generic predecessors.

### 3.2. Program Delivery Setting and Organization

With the exception of two programs [29,33], all others were delivered in an ambulatory outpatient setting, owing to their modular organization and duration over several weeks. One of the exceptions was a protocol for a future program (ParkProTrain) on physical activities: The program is to start during an intensive multimodal inpatient therapeutic program, but then to be continued independently at home for nine months with online sessions provided via an app [33]. One other study compared program delivery in small groups with home-based self-study [29]. For 5 programs (28%), a professional teaching manual was reported [25,26,28,29,32], for 4 (22%) teaching manuals and handouts to participants [24,30,32,34], and for 6 (33%) homework/home exercise programs in between single modules [22,30,31,32,35,37]. Only 2 programs (11%) explicitly report the integration of digital components [31,33]. As mentioned, one future program includes an app-based intervention at home [33]. Another program reports the provision of program-supporting information on a patient-orientated webpage [31].

### 3.3. Evaluation and Outcomes Measures

Both qualitative (3, 13%) [23,27,38] and (9, 39%) quantitative studies have been performed for evaluation of PD-related self-management programs [21,22,26,30,31,32,34,39,40]. Additionally, 8 (35%) studies combined quantitative data collection with qualitative descriptive aspects for program evaluation [24,28,29,36,41,42,43,44].

Of the quantitative studies, 3 reached level I according to AACPDM study quality scoring [32,39,44], 3 level II [29,31,40], 4 level III [21,26,36,43], 6 level IV [22,28,30,34,41,42], and 1 level V [24]. Controlled trials (10, 43%) compared to standard care [21,26,28,31,32,36,39,40,43,44], or compared two defined interventions (1, 4%) [29]. One additional study (1, 4%) compared two intensity levels of a multimodal intervention (SME plus physical/speech training) with usual care [32]. Selection criteria mostly were liberal, including PwPDs in different disease stages and with varying disease-related complications. Due to the nature of the interventions, none of the studies was blinded. 6 studies (26%) employed a delayed-start design, meaning that persons randomized to the control group received the intervention with a time delay [21,26,31,39,40,44]. Some programs restricted inclusion to certain disease stages (e.g., Hoehn and Yahr 2–3, or <4, for details see Table 1, Table 2 and Table 3). As mentioned, one study evaluated disease-stage specific programs and another program targeted PwPDs with major depression, both accompanied by corresponding study inclusion criteria [22,29].

Evaluation timepoints were at baseline and immediately or with little delay after the last element of respective intervention, and in 6 (26%) at varying intervals (2–12 months) after the intervention ended to assess for sustained effects [28,29,30,32,34,40].

Reported primary or secondary outcomes and employed measurement instruments fell into all domains of health-related outcomes [45], ranging from physiological/biological variables (e.g., neuroprotective markers, brain-derived growth factor (BDNF) [29] or multimodal brain imaging [35], over symptom status (e.g., Apathy Scale) [29], functional status (e.g., Berg Balance Score (BBS) [34], general health behaviors (e.g., Brief Cope Scale) [25] to overall (e.g., Short Form 36 (SF-36)) [26] or health-related quality of life (e.g., Parkinson’s Disease Questionnaire-39 (PDQ-39)) [31].

The 5 most often reported outcomes were: Health related and general quality of life (12 studies, 52%) [26,28,31,32,34,36,40,41,42,43,44], followed by depression [26,29,31,34,39,40,41,42,43,44], and aspects of self-management or self-efficacy [22,26,29,40], diverse functional mobility measures [22,30,34] and Unified Parkinson’s Disease Rating Scale (UPDRS) [29,30,31] (for all other outcomes, see Table 1 and Table 3).

Although all studies reported various baseline characteristics and other contextual variables (e.g., highest level of formal education or cognitive state), statements about possible predictors, mediators or confounders were reported in 4 studies only [26,34,36,39].

There was only one program (PEPP, “Person Education for PwPDs and their carers”) evaluated in transcultural multicenter studies in seven European countries [44]. One additional study in Canada compared two centers in their program performance [22].

There were 3 qualitative studies, all with the purpose of formative evaluation [23,27,38]. 8 of the quantitative summative evaluation studies included additional formative elements [24,28,29,36,41,42,43,44], in addition to one quantitative study with a formative focus [42]. Examples of reported formative outcomes are: group experience, comprehensiveness, perceived usefulness, satisfaction, content relevance; 2/3 future protocols plan to report formative outcomes [25,33], 2/3 in a mixed methods design with a combination of qualitative and quantitative study methods.

### 3.4. Efficacy and Effectiveness

Although only some of studies stated the primary outcome clearly or the study power (primary outcome: [40,44]; power: [21,36]; primary outcome and power: [29,32]), 11/15 (73%) reported an intended effect in any of the reported outcome variables [22,28,29,30,31,34,36,40,42,43,44]. Beneficial effects on (health-related) quality of life were reported in 6/11 (55%) studies that included any measurement instrument for quality of life. In addition, beneficial effects on the following outcomes were reported (for PwPDs): Any part of the UPDRS (2 studies) [30,31], depression (2) [29,34], self-management scales (1) [22], active problem-orientated coping (1) [40], fatigue (1) [34], diverse functional mobility measures (1) [22], general psychosocial burden (1) [42]; (for caregivers): mood (3) [28,39,44], burden (2) [28,44], relaxation techniques (1) [26]. 2/15 studies failed to report any effect on the respective outcome measures [21,41]. One had a small study group of 36 only [41], one used a measurement instrument (SF-12) for quality of life [21] that was not used in any other of the reported studies.

Although positive effects on any outcome measure could be found for up to 12 months post-intervention [34], most studies report an effect attrition in the post-intervention observation period ranging from 3 to 12 months. One study with a combined exercise/self-management support intervention reported effects on physical outcomes to be more stable than on psychosocial outcomes [34].

Given the fact that most studies were mono-central, small in study size and mostly carried out by the intervention teams themselves, they are not suited to assess effectiveness. However, the PEPP and its successors are those programmes in which the most independent evaluation studies were carried out, some of them by totally independent research teams in different countries [27,40], and one study explicitly targeted at assessing the reproducibility of effects after program transferal to clinical practice [28]. All these studies including the latter were able to reproduce some effect on the measured outcomes, so that this could be considered as evidence for general effectiveness of the PEPP and its off-springs.

### 3.5. Comparison to Other Systematic Reviews, Limitations

The search identified 5 other former systematic reviews on self-management interventions in PD (Table S2). One review was on general and disease-specific SME programs [46], while others focused on specific self-management programs for anxiety [47], falls [48], physical exercises [49] or occupational therapy-related interventions [50]. All reviews utilized the search terms “Parkinson’s” and “self-management” among others. From the 23 articles included in the current review, between 1 [47,48] and 6 [46] were also included in one of the five other identified systematic reviews. The relatively small overlap can mostly be explained by different eligibility/exclusion criteria.

One study met all inclusion criteria, but was not identified by the initial search terms [20] (Table 1). This study compared physiotherapy in a community-based setting with and without additional components for SME, whereas the absolute program intensity was kept stable in both arms. The study found an increased level of physical activity in the arm with SME, stable over two years. The study included several additional measures to increase adherence, such as a treatment contract, a personal logbook, feedback via sensors and a personalized webpage.

The finding of this additional study points to a potential limitation of the current systematic review. Due to the selected search terms sources could have been missed that use alternative terms for interventions targeting health-promoting behavioral modifications.

## 4. Swedish National Parkinson School (NPS)—Example of a Nationwide Implementation of a Self-Management Education Program for Persons with Parkinson’s Disease (PwPDs)

The Swedish National Parkinson School (NPS) has its origin in the “Person Education for PwPDs and their carers” (PEPP), developed by the European EduPark consortium in 2002 [41]. The Swedish NPS has been available and offered in clinical practice since 2014. In 2013 the PEPP was translated into Swedish and adapted to suit the Swedish healthcare services. The PEPP was chosen as a model for the NPS because it incorporated the ideas of a standardized and systematic self-management education that could be offered nationwide but still recognizing the major importance of an education that was person-centered and viewed patients and care partners as vital members active participants in their own care [42,51]. The PEPP had also been evaluated and found feasible and valuable for the participants and showed improvements in several outcomes for both PwPD and care partners [51]. Much of the content in the Swedish NPS s still similar to the original PEPP program but in the NPS there is an even greater emphasis on the importance of shared resources to handle life, and the Swedish NPS is provided entirely as a dyadic intervention. Development of the NPS was made in collaboration with representatives of healthcare professionals, PwPDs and their care partners and the pharmaceutical industry. The development of the NPS was undertaken as a project in clinical care and a detailed description of the translation and adaptation process is provided by Carlborg [52,53].

### 4.1. Program Description

**Figure 1:**
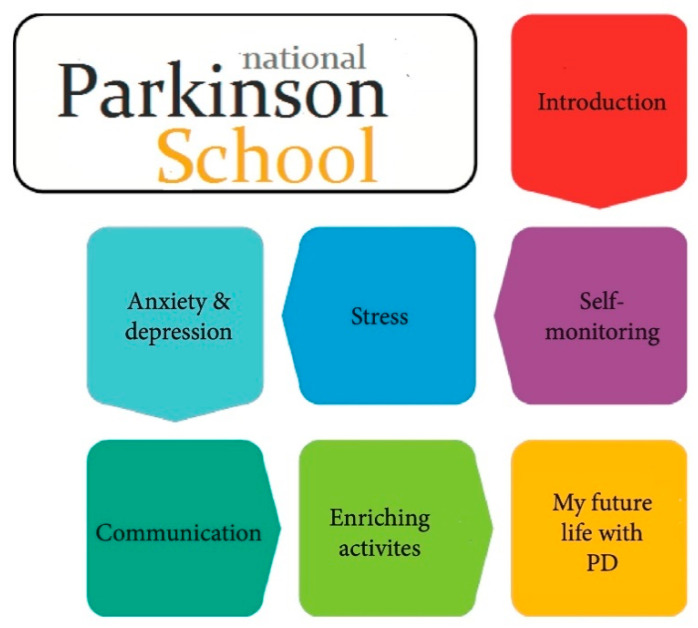
Modular elements of the Swedish National Parkinson School.

The PEPP is focusing on strategies to manage the psychological and social impact of the disease and the main goal is to empower participants to better deal with the challenges brought on by PD, primarily focusing on the psychological and social impact of the disease [42]. This is also kept as the main goal of the Swedish NPS as it aims to provide PwPDs and their care partners with the knowledge and cognitive strategies needed to improve their ability to manage the impact of PD in everyday life. The focus of the NPS is on the importance of a constructive and positive mindset and the skills needed to continue a fulfilling and satisfying life even in the presence of PD. This is done by enhancing awareness of own thoughts, feelings and actions in relation to the impact of PD on their everyday lives. How PwPDs and their care partners choose to relate to disease and their changing life situation greatly affects their ability to maintain good satisfaction with life, despite the difficulties. The introduction of techniques for self-monitoring and self-awareness included in the NPS gives participants the tools needed to initiate adaptation and the changes needed to reduce the impact of disease. The Swedish NPS program includes seven topics inspired by the structure of the PEPP [44]. These are: introduction with a focus to learn more about PD, self-monitoring, stress, anxiety and depression, communication, enriching activities, and my life with PD (Figure 1). One topic is in focus each time the group meets and each session is two hours long. During each session, the PwPDs and care partners meet in a small group of 12–14 participants. A certified educator, a health care professional with extensive experience of supporting PwPDs and their care partners, as well as with medical knowledge of PD, guides the group through the sessions. The educator has been trained to deliver the contents of the NPS and has been educated about the underlying concepts and aims of the program. Each session of the NPS program has a certain standardized structure, which begins with an introduction involving facts and information on a topic related to everyday life with PD. This is followed by group discussions relating to the information which has been presented. Group discussions focus on participants’ own experiences and thoughts, and provide an opportunity for peer learning and support found to be valuable and important by participants [42]. The new knowledge presented during the session is afterwards applied to the participants’ own life situation through practical exercises and home assignments, which are discussed and followed up during the next session. Each session of the NPS ends with a 15-min relaxation exercise.

### 4.2. Outlook and Evaluation

Approximately 20–30 groups undergo the NPS in clinical care in Sweden each year. The program has until now mainly been provided at neurological and geriatric clinics connected to the larger hospitals, which is due to the allocation of resources in the Swedish health care system. With improved and widespread technology like easy and available apps for video conferences, new ways of providing the NPS also to persons living in more remote areas are under development. Recent research shows that the NPS is considered valuable for the participants and contributes to enhanced adaptation and acceptance of life with PD as well as techniques for self-knowledge to manage symptoms of disease in everyday life. The dyadic approach was viewed as a shared platform of knowledge and understanding and the small group format exchanging experiences and feeling support from others in the same situation was highly appreciated [38]. After attending the NPS, PwPDs’ own perception of their health status was improved as well as their shift in approach to not letting the disease control life and their knowledge of strategies to handle symptoms [36]. The techniques of self-monitoring introduced in the NPS remained a strategy for PwPDs and care partners to explain and communicate health status in clinical encounters with the physician [27].

## 5. Concept for a Nationwide Structured Self-Management Education (SME) Program in Germany

Given the high importance of self-management for PwPDs and given the sometimes decade-long nationwide availability of certified SME programs for persons with similar diseases in Germany, PwPDs should have the same opportunity to benefit from the general availability of such a structured, evidence-based and quality-controlled program. In order for this to become reality, the same level of professional effort is needed that enabled the nationwide availability of programs for other comparable diseases.

In spite of the evidence for efficacy/effectiveness for a variety of existing programs for PwPDs worldwide, the effects were comparably small, not always reproducible and exposed to an important attrition effect often already 6-months post intervention (Table 1, and Section 3.4 above). Although most programs make some reference to the self-management concept, content and implementation strategies vary largely. The Swedish National Parkinson School is to our knowledge the only example of sustainable nationwide establishment of such a program, and for its basis (PEPP) there is arguably the most evidence for effectiveness.

However, existing programs should rather be understood as a valuable source of inspiration then as a ready-made solution that simply needs to be implemented broadly. Most concepts have never achieved the stage of broad sustainable implementation, or in the case of the PEPP were developed 18 years ago. It is very unlikely that a program developed in 2002 still provides the optimal answer to needs, expectations and best implementation strategies in the context of rapidly evolving living conditions and a disruptive digital transformation process taking place.

Any novel and future-orientated program can profit from the knowledge and experience generated through the various existing programs, but equally requires innovations concerning content, delivery concept including possible digital components, concerning a meaningful evaluation concept and to meet the regulatory requirements for a nationwide certification.

In the following sections, conceptual considerations about possible areas of improvement for a future-orientated and sustainable SME program are given. In addition, a sequential implementation concept is presented, consisting of 4 phases from a structured needs assessment of both PwPDs and experts to the final step of obtaining certification as the basis for nationwide implementation in Germany.

### 5.1. Conceptual Considerations

#### 5.1.1. Different Program Content and/or Delivery Concept Needed for Different Disease Stages?

One potential shortcoming is that there is no concept for a disease-stage adopted program. It is unclear when PwPDs are to be educated, whether this education should be repeated over the decade-long disease course, and if so, how much of the program needs to be adopted to possibly target several subpopulations. As described, in Canada such disease-stage-specific SME programs have been implemented, consisting of three subprograms: an early program for general disease management (EMP), an intermediate program for safe mobility (SMP), and a late-stage program for fall prevention (FPP) [22]. So far, only immediate post-intervention effects have been reported for the first of the three programs, targeting the early disease stage.

**Figure 2:**
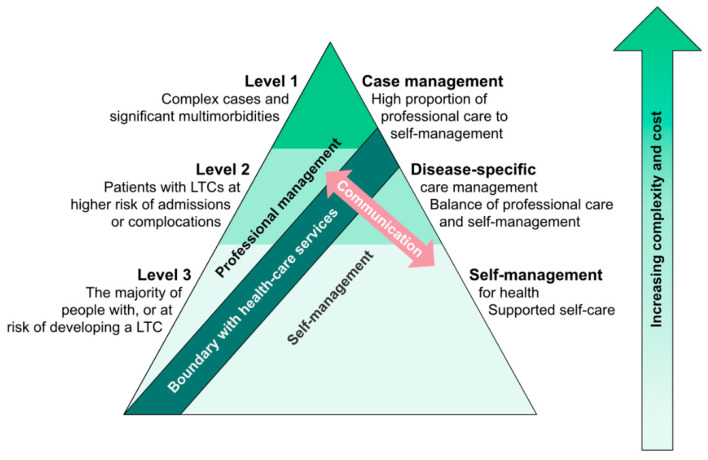
“Kaiser pyramid” about the interaction between patient disease-related self-management and professional management. In chronic diseases with little complexity in disease burden and/or therapeutic needs (level 3), self-management by persons with chronic conditions (PwCDs) is the major contributor to overall management, relative to the minor contribution of professional disease management. The more complex the disease and related therapeutic needs become (levels 2 and 1), the smaller becomes the relative contribution of self-management in comparison to professional management. The higher the relevance of professional management becomes, the more important becomes professional management support, e.g., by structured case management. Adopted from [8].

However, theoretical considerations about the varying requirements in self-management over the course of chronic diseases argue for this approach. In diseases like PD, both symptom load and treatment responsiveness vary strongly with time [54,55]. A conceptual pyramidal model developed by the managed care consortium Kaiser Permanente illustrates that the relative importance of self-management on the one side and professional disease management on the other is a function of the complexity of the therapeutic needs for a specific chronic disease: The less complex the needs are, the more affected persons can achieve through self-management independently, whereas with increasing complexity the relevance of professional medical disease management increases [56,57] (Figure 2).

PwPDs can be expected to wander along this “pyramid” during their disease course: In the honeymoon phase therapeutic needs are of lower complexity and the relative contribution of person self-management to overall disease management can be expected to be high [5,58]. In intermediate and early advanced diseases stages the therapeutic complexity increases sharply and with this the relative importance of professional disease management. In late disease stages, one could argue that the possible professional therapeutic contribution diminishes again, accompanied by an increase in the relative contribution of person self-management. Thus, even if it is arguable how well the “Kaiser pyramid” is applicable to PwPDs, it illustrates that the need in SME will vary importantly during the course of a sometimes decade-long chronic disease, both in content and possibly also in the delivery concept.

Another argument for a stage-specific program design is the importance of modeling and social persuasion for successful self-management interventions [3]. The better participants can relate to each other and the better the program is related to the participant’s contextual living situation, the easier it will be to achieve the intended behavioral changes. Regarding this, it is questionable how well PwPDs in early disease stages are to be expected to relate to PwPDs in advanced disease stages and with a much heavier affected living reality [59].

A third argument for stage-specific program is that cognitive capacities are inflicted along the disease course, with nearly all PwPDs to be expected to become demented in advanced disease stages [60]. Since all self-management programs follow the principles of cognitive behavioral therapy, not only the content, but also the content delivery should be adopted to varying cognitive capacities.

#### 5.1.2. Importance of Disease-Related Information and Knowledge Skills

The PEPP has a strong strategic focus on the CBT-based self-management intervention and dedicates the majority of the program to teaching behavioral skills such as action planning, self-reflection, reframing, self-efficacy beliefs or relaxation techniques [41]. Relatively little of the time is reserved for disease-related information. The focus is rather on the skill of self-dependent retrieval of high-quality information from public information resources than on actual information provision. However, given the complexity of the disease, of medication-induced complications and of the high importance of informed self-monitoring, it is questionable whether teaching relevant knowledge should not be strengthened without weakening the efforts in SME. The importance of actual knowledge transfer is also fueled by the limited availability of cost-free high-quality person-orientated information offers.

#### 5.1.3. Digitalization as a Promising Measure against Effect Attrition

The effects of all SME programs are exposed to attrition [8]. Even if this lies in the nature of all behavioral interventions, the ambition to achieve measurable effects for time periods longer than 6 months appears reasonable. Therefore, it is important to consider strategies to consolidate the newly acquainted behavioral competences. Previous studies have already postulated the need of a “booster effect” [28].

In addition, any future program needs to be cost-effective to achieve financing agreements with statutory health insurers as the only realistic long-term financer in Germany. Continuous disease-accompanying SME will most likely neither be feasible nor financeable. Therefore, complementing SME with a digital and possibly long-term follow-up intervention appears as the only realistic option to promote more sustainable effects than with the current and mostly purely analogue programs.

The potential of a possible digital extension is illustrated by the growing body of evidence for the effectiveness of online behavioral cognitive therapy, including for elderly PwCDs [61]. In addition, results from a preliminary focus group on PwPDs’ needs revealed an acceptance of online supplementary elements.

If the goal of any future SME program is to assure equal access, than a digitalized automated program element is likely the most effective measure to achieve this.

#### 5.1.4. Combination with Related Therapeutic Interventions

Many of the preceding programs combined SME with other therapeutic interventions, such as physiotherapy or occupational therapy. It is unclear whether SME programs are more or less effective when combined with other interventions, in spite of single studies addressing this question [29].

However, such a combination appears as a promising concept: If self-management relies on the mastery of skills needed for induction and maintenance of health-promoting behavioral change and if domain-specific training (modeling) of these skills is an important strategy [3,17], then the combination with therapeutic interventions in domains such as physical activity appears as a synergic approach.

#### 5.1.5. Evaluation Concepts for SME Programs

As mentioned, in a majority of studies distal complex outcomes such as QoL have been reported as primary outcome, although only an indirect effect through mediators and/or more proximal outcomes can be postulated. This provides one possible explanation for the often small effects in combination with relatively rapid effects attrition.

The importance of a careful study design has been recently illustrated by a recent Swedish quasi-experimental case-control study on the NPS [36]. Although some beneficial effects on QoL could be detected immediately after the intervention by within-group comparisons, effects on other outcome variables only became apparent by longitudinal between-group comparisons: Whereas satisfaction with life as a whole decreased in the control group, it remained stable in the intervention group, an effect that would have been missed without a longitudinal between group comparisons. Several studies have considered the importance of possible confounders, effect moderators or mediators, but have failed to illustrate an impact of any of the studied constructs [36,39]. Mostly no rationale is given for the relative importance of the constructs considered as possible confounders and/or mediators, and the respective studies do not report on their power to illustrate the postulated effects and, therefore, have to be considered exploratory.

**Figure 3:**
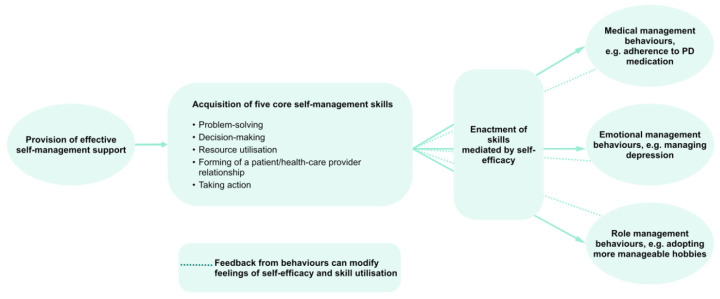
Structural effects model for SME interventions according to the theory of behavioral change. Adopted from [5].

In order to avoid the sole reliance on distal complex outcomes such as QoL, exposed to a multitude of effects besides the SME program to be evaluated, evaluation concepts should consider the theoretical basis of SME programs. As mentioned, the starting point of numerous SME programs is the Chronic Disease Self-Management Program (CDSMP) that is based on Bandura’s theory of behavioral change, validated in numerous studies not only on health-related, but also on general behaviors [14,62]. Only if the underlying cascade of Bandura’s theory, ranging from skills over self-efficacy beliefs to self-management behaviors, is functional, can an effect on distal outcomes such as QoL be expected. To understand how effects are transmitted through this cascade, evaluation concepts should measure not only distal outcomes, but all intermediate constructs and their dependencies as established by the theory of behavioral change (Figure 3). Considering that self-management behaviors are the primary target of SME programs, there is a strong rationale for not defining QoL as the primary outcome, but self-management behaviors and their (sustained) improvement.

### 5.2. Sequential Design and Implementation Concept for a Certified Nationwide SME Program for PwPDs

**Figure 4:**
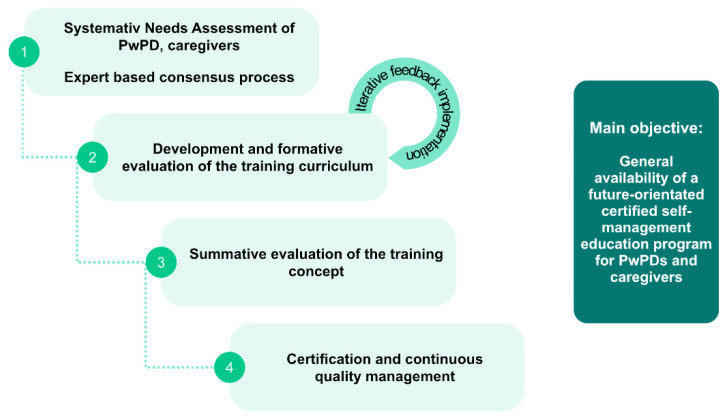
Sequential phases suggested for needs assessment (phase 1), design (phase 2), evaluation for effectiveness (phase 3), and for certification and nationwide implementation of a structured SME program for PwPDs (phase 4).

To address the above mentioned issues and to achieve the goal of a nationwide certified PwPD SME program, the following sequential phases are suggested (Figure 4):

(1) Phase 1: Systematic needs assessment of PwPDs and caregivers in combination with an expert-based consensus about contents, delivery concepts and program objectives;
(2) Phase 2: Development of an SME program in an dynamic co-design process, including formative evaluation;
(3) Phase 3: Proof of efficacy and proof of effectiveness in a multicenter setting, representative of the later application context;
(4) Phase 4: Obtain certification and agreement of funding with statutory health insurers.

#### 5.2.1. Phase 1: Systematic Assessment of Needs of PwPDs and Caregivers and Expert-Based Consensus Process

**Figure 5:**
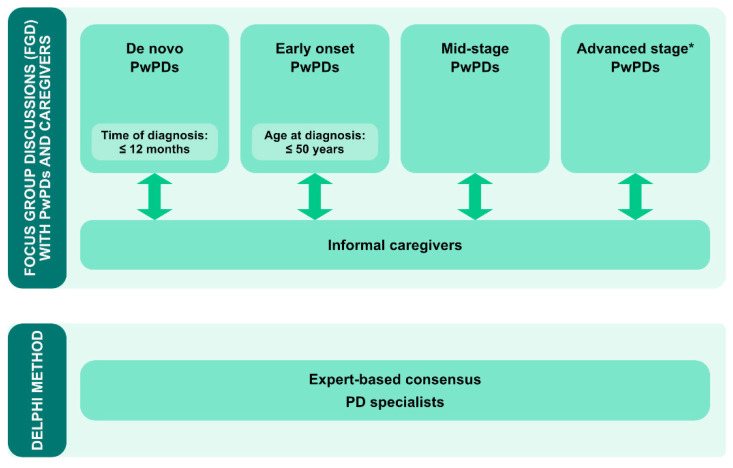
Concept for phase 1: structured assessment of needs of PwPDs and their caregivers by disease-stage specific focus groups (upper panel). Structured expert-based Delphi consensus process on expert recommendations on content, format and objectives of a PD-specific PME program. * Advanced disease stage will be defined according to [58].

As a first step, the needs of both PwPDs and their caregivers are to be systematically analyzed (Figure 5). Both groups should be iteratively involved along the entire development and evaluation phase to assure patient and caregiver engagement due to constant interaction with the program design team.

To understand expected disease-stage specific needs from the beginning of the process, four different person subgroups are to be assessed separately for their specific needs in a structured format, e.g., focus groups: (1) de-novo PwPDs (defined by time since diagnosis ≤12 months); (2) early onset PwPDs (≤50 years); (3) general early to mid-stage PwPDs (defined as PwPDs not fulfilling the criteria for the subgroups 1,2 and 4), and (4) PwPDs with advanced disease stages (defined by consented criteria for advanced PD [58]. This differential approach is deemed necessary to understand disease-stage specific needs in enough detail for the design of disease-stage specific components. This holds especially true for young PwPDs and their caregivers, as this group typically faces very distinct self-management challenges (e.g., underaged children, professions) compared to older PwPDs [59].

Focus groups will be distributed in a geographically representative manner over Germany to consider a potential impact of regional region cultural contexts. Simultaneous, but separate, focus groups for caregivers will be organized to assess their specific needs, potentially distinct from the needs of PwPDs. Two focus groups with PwPDs and caregivers each were already administered as a pilot.

This person-centered needs assessment will be complemented by an expert-based Delphi consensus process to obtain recommendations and expectations of PD specialists both about content and delivery format [63] (Figure 5). Experts from several PD-related sub-specializations will be approached, such as specialists for deep brain stimulation (DBS), continuous treatment options or non-pharmacologic treatment options. Besides gathering expert advice, the structured assessment of the experts’ perspective should help to assure professional acceptance, which is an important success criterion for the later program.

Overall, the results from the focus group discussions and the Delphi consensus will constitute the knowledge base for a person-centered concept and design phase to follow.

#### 5.2.2. Phase 2: Program Development and Formative Evaluation

**Figure 6:**
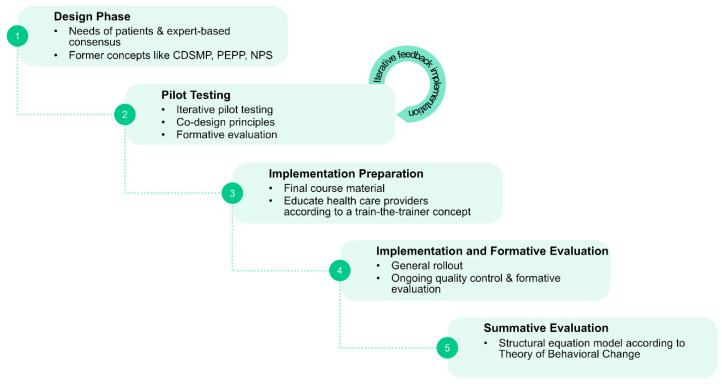
Sequential steps in phase 2.

Based on the comprehensive needs assessment and on the experiences with the existing programs, a new SME program needs to be developed (Figure 6). How much of the former programs can be overtaken will depend on the directions given in the needs assessments concerning the core questions as formulated above: e.g., whether and if how many disease-stage specific components are needed; what importance should be given to disease-related knowledge transfer; or what degree and what strategy is most promising to integrate digital components to a future program. The design process should follow iterative co-design principles to assure that new developments are constantly evaluated for their appropriateness from the target group’s perspective [64]. Adherence to concepts for agile product development is to assure that several iterative rounds of developing, testing and evaluation can be carried out. Both qualitative and quantitative formative evaluation methods will be applied.

#### 5.2.3. Phase 3: Summative Evaluation of the New Concept for Efficacy and Effectiveness

As soon as the new program has achieved a sufficient maturity according to the repetitive formative testing, it will be assessed for efficacy in an adequately sized randomized controlled pilot trial. The evaluation concept should be in compliance with the effects model behind the theory of behavioral change and will comprise measurement instruments for all relevant constructs, as described.

After efficacy has successfully been established, a multicenter trial is to follow to illustrate effectiveness in a real-world scenario. For this to be achieved, not only the quality and maturity of the new SME program itself will be relevant, but also the design of adequate related management and quality control mechanisms, e.g., the schooling concept for the effectuating healthcare providers and a concept to assure sufficient procedural program adherence in diverse implementation settings.

#### 5.2.4. Phase 4: Program Certification in Compliance with German Legislative and Institutional Regulations

To implement an SME program into the regular care process in Germany, developers have to address several requirements in a standardized process on quality, structure, outcome and content of the program [65].

The association of statutory health insurers in Germany developed “joined recommendations for the promotion and implementation of patient education programs” as early as 2001 (recently updated) to assist more than 100 statutory health insurances in Germany in the evaluation of SME programs [66]. The main criteria is the overall effectiveness [67].

#### 5.2.5. Legal Foundations

The German Social Insurance Code (SGB V) regulates the legal basis for the promotion and implementation of SME programs for persons with chronic long-term conditions.

Before approving a specific SME program, an insurer has to inspect the evidence for effectiveness. Expert opinion can be requested from the “Medizinischer Dienst der Krankenkassen (MDK)”, a service unit developed jointly by the statutory health insurances to control medical care processes. To be formally assessed, information has to be submitted on the structure and content of the program together with information about its effectiveness, local facilities and proof of qualification of the interdisciplinary educational team [67] (Table 4). This inspection process has to be completed before program inscription starts.

Applicants receive a non-negotiable approval or refusal note. Approval comes with a valid certificate, but the specific reimbursement still has to be negotiated with each of the statutory health insurers separately. Even though this process is heavily regulated, strengths are the standardized and transparent requirements, offering equal chances to all applicants.

Reimbursement can be granted if the insured person recently received medical treatment for a specific disease or is currently proceeding such a treatment and if the participation rate was >80% [66]. Caregivers are to be involved if indicated by medical reasons.

## 6. Discussion

Despite the numerous arguments for a structured SME program for PwPDs and despite the fact that persons with similar chronic diseases (e.g., diabetes, asthma) have access to such programs as part of the regular catalogue of benefits of statutory health insurers, so far this could not be achieved for PwPDs in Germany. To our knowledge, The NPS in Sweden is the only example of nationwide coverage with an SME program for PwPDs.

However, there are many arguments why this should not be the exception but the norm. It is widely accepted that integrated care concepts should be person-centered [5,69]. Therefore, probably the most important argument is that PwPDs repetitively state that self-management support is their top priority in the context of integrated care approaches [6,7]. In addition, PwPDs can be expected to face comparatively high challenges in self-management, given the multidimensionality of symptoms and the high complexity of the therapeutic measurements. If self-management behaviors substantially contribute to health-related outcomes according to theoretical considerations (e.g., “Kaiser pyramid” [57]), both PwPDs’ and experts’ assumptions [7,16] and clinical evidence from various programs, then one could conclude that self-management education should be one of the core elements of any integrated care concept for PwPDs.

Person-centered care considers the needs of all persons involved in a care relationship—be it the patient, informal or formal healthcare providers—to be of equal importance [70]. SME is in this context an effective measurement to promote person-centered care because it does not only address the needs of patients but also the needs of healthcare providers to profit from self-management-competent patients in their efforts to provide optimal care.

As mentioned, there are only few reported studies on temporary regional efforts in structured SME for PwPDs in Germany [28,40,42,51]. However, this does not mean that there currently is no self-management support—only that it is mostly provided in an informal manner and without standardized professional education on how to deliver it. Most likely, many healthcare professionals are not aware that they have integrated self-management support into their personal strategy of caring for PwPDs. Such informal self-management support does not facilitate the assurance of equal access independent of socioeconomic patient background or the implementation of measurements for ongoing quality monitoring and improvement.

Leaving self-management support to the discretion and personal capacities of individual healthcare providers is not compatible with the ambition to offer more equal, timely and better quality care through structured integrated care concepts. Whereas some components of integrated care concepts need to be tailored to the specific regional implementation context, SME programs do not belong to these. Rather, basic principles and assumptions have been repetitively shown to be valid across different diseases and implementation settings, including transcultural implementation [16].

Taking into consideration the diverse measurements to be undertaken to arrive at a truly future-orientated sustainable SME concept, it appears reasonable to not develop such a program for each integrated care concept separately, but jointly in a collaborative effort. Not only will such an approach be more economic, but several reasons regarding both content and delivery concept speak in favor for a collaborative effort: a person-centered self-management program should profit from a broad spectrum of expert experiences and knowledge, which in turn represents the basis for reaching a sustainable professional consensus and acceptance on content and structure of a future program; both an agile person-centered iterative design process and the development of supporting innovative digital components for such a future-orientated program require significant resources that can better be assured in a joint effort than in several smaller initiatives; ongoing formative and summative evaluation, especially for general program effectiveness, will be facilitated by a mutually agreed evaluation concept and set of measurement instruments.

Last but not least, such a collaborative effort will be the best argument to convince statutory health insurers that PwPDs not only have the same rights as persons with other chronic diseases to benefit from the offer of a structured SME program, but that there also is a high-quality concept available worth being funded, supported both by a strong professional commitment and by a substantial body of evidence for program effectiveness.

The outlined steps to achieve this goal can only be addressed with sufficient resources. For this, public funding needs to be assured through a grant proposal, currently under preparation. Professionals or institutions interested in contributing to this effort, e.g., as a clinical partner, as an expert in person-centered co-design processes, in the evaluation of complex healthcare interventions, or in the design of digitally supported cognitive behavioral therapy and/or SME programs are invited to contact the authors.

## Figures and Tables

**Table 1:** Quantitative evaluation studies.

**Author, Date, Country**	**Program**	**Study Goals**	**Study Design/** **Population**	**Intervention Content**			**Intervention Format**	**Measurement Instruments** (BL/OTH (baseline, others (e.g., possible confounder)), PO (primary outcome), SO (secondary outcome), O (outcome not defined) **Evaluation Timepoints**	**Outcome,** **Evidence Level (AACPDM)**
				**Information Provision**	**Behavioral Modification**	**Physical Exercises**			
A’Campo et al., 2009, Netherlands	EduPark/PEEP *Patient Education Program for Parkinson’s disease*	(1) evaluation of effectiveness of PEPP	RCT, monocenter pre/post-test design additional formative evaluation *intervention group* PwPD (*n* = 35) CG (*n* = 26) *control group* PwPD (*n* = 29) CG (*n* = 20) *comments:* sample size based on feasibility	health promotion, stress management, management of anxiety/depression, role of unrealistic, unhelpful cognitions, ways of communication	based on behavioral cognitive therapy, importance of taking active/central role in health care system, self-monitoring techniques (using a diary for fluctuation of symptoms), social competence and support	body awareness (breathing, muscular tensions), relaxation exercises	*intervention group* 8 wk PEEP 8 interactive group sessions (1 per wk, 90 min, 5–7 participants) active information, exercises, homework, video clips, role plays professional trainers (2-days training workshop) standardized manual (6 languages) CG: simultaneous separate sessions *control group* usual neurological care delayed start design: intervention after last observation	*quantitative* HandY (Hoehn and Yahr Scale), BL/OTH, t_0_ (PT) MMSE (Mini Mental State Examination), BL/OTH, t_0_ (PT, CG) ADL (Activities of Daily Living Scale), BL/OTH, t_0_ (PT) sociodemographic data, BL/OTH, t_0_ (PT, CG) mood scale (100-point VAS), BL/OTH, t_1_, (PT, CG) EuroQol-5D, ∆PO (subscales VAS, utility) (CG) SDS (Self-rating Depression Scale), ∆PO, t_0_, t_2_ BELA-P-k (Belastungsfragebogen Parkinson Kurzversion), ∆PO (“bothered by”, “need for help” score), ∆SO (subscales), t_0_, t_2_ BELA-A-k (Belastungsfragebogen Parkinson Angehörige Kurzversion), ∆PO (“bothered by”, “need for help” score), ∆SO (subscales), t_0_, t_2_ PDQ-39 (Parkinson’s Disease Questionnaire-39), ∆PO (SI), ∆SO (subscales) (PT), t_0_, t_2_ *descriptive* evaluation questionnaire, t_2_ *evaluation timepoints* t_0_ = baseline, 2 wk before PEEP, t_1_ = before and after each session, t_2_ = 9 wk after beginning of PEEP	*baseline* ↓↑ differences between groups ↓ MMSE score (intervention group) *PT* ↑ mood scale ↓↑ effects in patient scores (↓) PDQ-SI in intervention group *CG* ↓ BELA-A-k total ↓ BELA-A-k subscores: “achievement capability”, “emotional functioning”, “social functioning” *descriptive* helpful exchange of experiences improvement of understanding of PD and deal with problems stress management most valued session *evidence level* I
A’Campo et al., 2012, Netherlands		(1) secondary analysis of RCT for potential effect modifiers (A’Campo et al., 2009)							*linear regression analyses* MMSE (PT) predicts BELA-A-k subscore “bothered by” (CG) no modifiers for PT *evidence level* *I*
A’Campo et al., 2011 Netherlands	EduPark/PEEP *Patient Education Program for Parkinson’s disease*	(1) evaluation for effectiveness of PEEP in daily clinical practice without controlled academic conditions (2) comparison with previous RCT (A’Campo et al., 2009) (3) assessment of effectiveness at 6-mth-follow-up	non-randomized controlled design (historical control group), pre-test/post-test design, additional formative evaluation *intervention group* PwPD (*n* = 55) CG (*n* = 50) *control group* PwPD (*n* = 35) CG (*n* = 26) *comments:* clinical practice groups compared with RCT groups (A’Campo et al., 2009)	health promotion, stress management, management of anxiety/depression, role of unrealistic, unhelpful cognitions, ways of communication	based on behavioral cognitive therapy, importance of taking active/central role in health care system, self-monitoring techniques (using a diary for fluctuation of symptoms), social competence and support	body awareness (breathing, muscular tensions), relaxation exercises	*intervention group* 8 wk PEEP 8 interactive group sessions (1 per wk, 90 min, 5–7 participants) active information, exercises, homework, video clips, role plays professional trainers (2-days training workshop) standardized manual (6 languages) CG: simultaneous separate sessions *historical control group* usual neurological care delayed start design: intervention after last observation	*quantitative* HandY, BL/OTH, t_0_ (PT) MMSE, BL/OTH, t_0_ (PT, CG) ADL, BL/OTH, t_0_ (PT) sociodemographic data, BL/OTH, t_0_ (PT, CG) mood scale (100-point VAS), BL/OTH, t_1,_ (PT, CG) PDQ-39, PO, BL/OTH, t_0_, t_2,_ t_3_ (PT) BELA-A-k, PO, BL/OTH, t_0_, t_2,_ t_3_ (CG) *descriptive* evaluation questionnaire, t_2_, t_3_ *evaluation timepoints* t_0_ = baseline, t_1_ = before and after each session, t_2_ = 9 wks after beginning of PEEP, t_3_ = 6 mth follow-up	*baseline* ↑ PDQ-39-SI (intervention group) *drop-outs* ↑ PDQ-39-SI ↓ BELA-A-k (subscale: “bothered by”) *short term effects (t_2_)* ↑ mood scale (PT, CG) ↓↑ PT and CG ↓↑ intervention and control group ↓ BELA-A-k ↓ PDQ-39-SI *descriptive* better QoL after participation (PT) less psychosocial burden, need for help (CG) *effects 6-mth-follow-up (t_3_)* ↓↑ baseline and follow-up (PT, CG) *descriptive* need for follow-up session (45% PT, 70% CG) 70% benefited from PEEP (PT, CG) improvement of communication less use of learned coping strategies *evidence level* IV
Chlond et al., 2016 Germany		(1) re-evaluate the effectiveness of PEEP among German PwPD (2) assessment of sustainability of effect (3) define the time when a booster session is needed to maintain long-term efficacy	RCT, multicenter pre-test/post-test design *intervention group* PwPD (*n* = 39) *control group* PwPD (*n* = 34) no CG				*intervention group* 8 wk PEEP 8 interactive group sessions (1 per 2 wks, 90 min, 5–7 participants) active information, exercises, homework, video clips, role plays professional trainers (two-days training workshop) standardized manual (6 languages) *control group* usual neurological care delayed start design: Intervention after last observation	MMSE, BL/OTH, t_0_ sociodemographic data, BL/OTH, t_0_ PDQ-39, PO, t_0_, t_1_, t_2_ Euroqol-5D, SO, t_0_, t_1_, t_2_ FKV-LIS-SE (Freiburg Coping with Disease Questionnaire), SO, t_0_, t_1_, t_2_ BELA-P-k, SO, t_0_, t_1_, t_2_ SOC-29 (Sense of Coherence Scale), SO, t_0_, t_1_, t_2_ GSE (General Self-Efficacy Scale), SO, t_0_, t_1_, t_2_ HAS-D (German Hospital Anxiety and Depression Scale), SO, t_0_, t_1_, t_2_ *evaluation timepoints* t_0_ = baseline, t_1_ = right after PEEP, t_2_ = 3 mth follow-up	*baseline* ↓↑ differences between groups *after intervention and follow-up (t_1,2_)* ↑ FKV-LIS-SE subscale (active problem-oriented coping) ↓↑ EQ-5D, BELA-P-k, SOC-29, GSE ↓ PDQ-39-SI ↓ PDQ-39 subscales (mobility, stigma, social support, bodily discomfort) *after intervention (t_1_)* (↑) EQ-5D VAS among intervention group, returned to baseline at follow-up *evidence level* II
Macht et al., 2007 Germany, Estonia, Finland, Italy, Netherlands, Spain, UK	EduPark/PEEP *Patient Education Program for Parkinson’s disease*	(1) patient-related formative evaluation of usefulness, comprehensibility and feasibility (2) describing measures applicable for a formative evaluation with sample of 7 countries	single group design, multicenter, pre/post-test design, formative evaluation PwPD (*n* = 150) no CG	health promotion, stress management, management of anxiety/depression, role of unrealistic, unhelpful cognitions, ways of communication	based on behavioral cognitive therapy, importance of taking active/central role in health care system, self-monitoring techniques (using a diary for fluctuation of symptoms), social competence and support	body awareness (breathing, muscular tensions), relaxation exercises	8 wk PEEP 8 interactive group sessions (1 per wk, 90 min, 5–7 participants) active information, exercises, homework, video clips, role plays, handouts, diary sheets trained psychologist (two-days training workshop) standardized manual (6 languages)	*quantitative (PO not defined)* MMSE, BL/OTH, t_0_ HandY, BL/OTH, t_0_ sociodemographic data, BL/OTH, t_0_ UPDRS part I and II, BL/OTH, mood scale (100-point VAS), BL/OTH, t_1_ activities of daily living scale by Schwab and England, BL/OTH, t_1_ PDQ-39, O, BL/OTH t_0_, t_2_ BELA-P-k, O, BL/OTH t_0_, t_2_ SDS, O t_0_, t_2_ descriptive evaluation questionnaire (opinion of overall session/program, provided information, learned skills), t_1,_ t_2_ *evaluation timepoints* t_0_ = baseline, t_1_ = before/after each session, t_2_ = after 10 wk	*baseline* ↓↑ homogenous patient characteristics across countries *post intervention effects (after each session)* ↑ mood scale ↓↑ PDQ-39, SDS ↓ BELA-P-k dDescriptive * intervention was appropriate and fulfilled expectations (67–80%) participants would recommend this program or participate in a similar nature new and helpful information exchange of experiences within the group was helpful improvement of understanding of PD (2/3) *evidence level* IV
Simons et al., 2006 UK		(1) description program elements (2) formative evaluation with sample of British participants (3) suggestion of recommendations for future implementation	single group design, pre/post-test design, additional formative evaluation PwPD (*n* = 22) CG (*n* = 14)					*quantitative (PO not defined)* MMSE, BL/OTH, t_0_ HandY, BL/OTH, t_0_ sociodemographic data, BL/OTH, t_0_ UPDRS part I and II, BL/OTH, t_0_ activities of daily living scale by Schwab and England, BL/OTH, t_0_ mood scale (100-point VAS), BL/OTH, t_1_ PDQ-39, O, BL/OTH t_0_, t_2,_ (PT) BELA-P, O, BL/OTH t_0_, t_2,_ (PT) BELA-A, O, BL/OTH, t_0_, t_2_ (CG) EuroQol-5D, O, t_0_, t_2_ (CG) SDS, O t_0_, t_2_ (PT, CG) *descriptive* evaluation questionnaire (opinion of overall session/program, provided information, learned skills), t_1,_ t_2_ *evaluation timepoints* t_0_ = baseline, t_1_ = before/after each session, t_2_ = after 10 wk	*post intervention effects (after each sessions)* ↑ mood scale (except of 2 sessions) (PT, CG) ↓↑ PDQ-39, BELA-P (PT) ↓↑ EuroQol-5D, BELA-A (CG) (↓) subscales BELA-P (PT) dDescriptive * participants received helpful information (agreement 50–100%) exchange of experiences within the group was helpful (agreement 72–100%) improvement ability to handle problems related to PD (agreement 78%) most useful session: stress management (50%) *evidence level* IV
Tiihonen et al., 2008 Finland	EduPark/PEEP *Patient Education Program for Parkinson’s disease*	(1) evaluation of effectiveness and applicability of PEEP in Finland	non-randomized controlled design pre/post-test design 2 centers *intervention group* PwPD (*n* = 29) HandY = 1–3 location: Turku *control group* PwPD (*n* = 23) HandY = 1–3 location: Helsinki no CG	health promotion, stress management, management of anxiety/depression, role of unrealistic, unhelpful cognitions, ways of communication	based on behavioral cognitive therapy, importance of taking active/central role in health care system, self-monitoring techniques (using a diary for fluctuation of symptoms), social competence and support	body awareness (breathing, muscular tensions), relaxation exercises	*intervention group* 8 wk PEEP 8 interactive group sessions (1 per wk, 90 min, 5–7 participants) active information, exercises, homework, video clips, role plays, handouts, diary sheets trained psychologist (two-days training workshop) standardized manual (6 languages) *control group* standard care	*quantitative (PO not defined)* MMSE, BL/OTH, t_0_ HandY, BL/OTH, t_0_ sociodemographic data, BL/OTH, t_0_ mood scale (100-point VAS), BL/OTH, t_1_ PDQ-39, O, BL/OTH t_0_, t_2_ BELA-P-k, O, BL/OTH t_0_, t_2_ ADL scale of UPDRS, O, BL/OTH, t_0_, t_2_ SDS, O t_0_, t_2_ *descriptive* evaluation questionnaire *evaluation timepoints* t_0_ = baseline, t_1_ = before and after each session, t_2_ = after 10 wks	*baseline* ↑ longer disease duration in control group *post intervention effects (after each session)* ↑ mood scale ↓↑ SDS without covariate adjustment: ↓↑ ADL scale ↓↑ BELA-P-k ↓↑ PDQ-39-SI (intervention group) ↑ PDQ-39-SI (control group) with covariate adjustment (years since diagnosis): ↓ PDQ-39 subscale (“Social support”) *evidence level* III
Tickle-Degnen et al., 2010 USA	self-management rehabilitation	(1) determine if self-management rehabilitation promoted HRQOL beyond best medical therapy (2) does more intense individualized rehabilitation increase effectiveness (3) persistence of outcomes at 2- and 6-months follow-up (4) Are rehabilitation-targeted domains (mobility, communication, activities of daily living) more responsive to intervention than non-targeted areas (emotions, stigma, social support, cognitive ability)?	RCT, monocenter *intervention group* *18 hrs rehabilitation* PwPD (*n* = 37), HandY = 2–3 *27 hrs rehabilitation* PwPD (*n* = 39), HandY = 2–3 *control group* *0 hrs rehabilitation* PwPD (*n* = 41), HandY = 2–3 no CG *comments:* power >0.80 (difference between rehabilitation and no rehabilitation)	no PD-specific content	assessing problems in personally valued domains of mobility, communication and daily life activities, observe behavior, identify strengths and problems in mobility, communication and activities of daily living, goal setting and implementation of action plans	physical and speech exercises, functional training	*intervention group* 6 wks of self-management rehabilitation (1) 18 hrs clinic group sessions (2 per wk, 1.5 h, 4 participants) and student-facilitated social group session in the clinic OR (2) 27 h clinic group sessions (2 per wk, 1.5 h, 4 participants) and a transfer-of-training session at home (1 per wk, 1.5 h) trained, supervised interdisciplinary team of physical, occupational therapists and speech therapists standardized and manualized handouts with photographs of exercise routine *control group* handouts with photographs of exercise routine	MMSE, BL/OTH, t_0_ HandY, BL/OTH, t_0_ GDS (Geriatric Depression Scale), BL/OTH, t_0_ PDQ-39, PO, BL/OTH, t_0_, t_1_, t_2_, t_3_ *evaluation timepoints* t_0_ = baseline, t_1_ = post intervention, 6 wks, t_2_ = 2-month-follow-up, t_3_ = 6-month-follow-up	*baseline* ↓↑ differences between groups ↑ PDQ-39 social support (0 hrs rehabilitation) *comparison rehabilitation* vs. *no rehabilitation* ↓ PDQ-39-SI (reduction of problems) ↓ PDQ-39 subscales (communication, mobility, activities of daily living) ↓strongest effect PDQ-39 subscale communication (2-month follow-up) ↓ strongest effect PDQ-39 subscale mobility (6-month follow-up) ↓↑ no differences in PDQ-39 between 18 h and 27 h intensities *evidence level* I
Guo et al., 2009 China	personal rehabilitation program	(1) development of a program with group education and personal rehabilitation focusing on HR-QOL improvement (2) empower people with PD to deal with disease-related challenges	RCT, single-blind, pre/post-test design, quasi-experimental, monocenter *intervention group* PwPD (*n* = 23), HandY = 1–3 *control group* PwPD (*n* = 21), HandY = 1–3 no CG	specific nutrition, antidepressant and anxiolytic medications, psychotherapy	management of daily disease-impacted problems	physical and tailored occupational therapy (e.g., balance training, active music therapy), practical exercise at home	*intervention group* 8 wks personal rehabilitation program 3 interactive group sessions (45 min) 24 personal rehabilitation sessions (30 min) multidisciplinary team (occupational therapist, physiotherapist psychologist, nurse, neurologist, dietitian) additional information on a website *control group* standard care, one session after end of observation period	MMSE, BL/OTH, t_0_ HandY, BL/OTH, t_0_ sociodemographic data, BL/OTH, t_0_ PDQ–39, PO, BL/OTH, t_0_, t_1_, t_2_ UPDRS part II, III, SO, BL/OTH, t_0_, t_2_ SEADL (Schwab and England ADL scale), SO, BL/OTH, t_0_, t_2_ SDS (Zung Self-Rating Depression Scale), SO, BL/OTH, t_0_, t_2_ PMS (Global patient’s mood status) SO, BL/OTH, t_0_, t_2_ CMS (Caregiver mood status), SO, BL/OTH, t_0_, t_2_ *evaluation timepoints* t_0_ = baseline, t_1_ = after 4 wks, t_2_ = after intervention (8 wks)	*baseline* ↓↑ differences between groups *after 4 wks* ↓ PDQ-39 subscale bodily discomfort *after 8 wks* ↑ PMS ↓↑ SEADL ↓↑ SDS ↓ PDQ-39-SI ↓ UPDRS part II and III *evidence level* II
Sajatovic et al., 2017 USA	EXCEED (exercise therapy for PD + CDSM group program)	(1) compare an individual versus group exercise plus CDSM program (2) acceptance and adherence of these programs (3) alteration of depression and factors of neural health and inflammation after these interventions	prospective RCT, monocenter additional formative evaluation *EXCEED intervention* PwPD + comorbid depression (*n* = 15), HandY = 1–3 MADRS ≥ 14 *SGE intervention* PwPD + comorbid depression (*n* = 15), HandY = 1–3 MADRS ≥ 14 no CG *comments:* power >0.80 (MARDS)	CDSM information, PD-specific content (not further described)	based on self-management approach, problem identification and goal setting	fast-paced, low-resistance cycling (20 min), strength training (20 min), progressive sequence of resistance bands	12 wks EXCEED CDSM group intervention (1 per wk, 1 h, 7–8 participants) nurse and trained peer educator with PD-Dep manualized sessions 3 times/week small group exercises with certified personal trainer detailed instruction manual after 12 wks participants continued to exercise on their own	*quantitative (O defined as exploratory outcome))* MMSE, BL/OTH, t_0_ HandY, BL/OTH, t_0_ sociodemographic data, BL/OTH, t_0_ CCI (Charlson Comorbidity Index), BL/OTH, t_0_ MADRS (Montgomery-Asberg Depression Rating Scale), BL/OTH, ∆PO, t_0_, t_1_, t_2_ MoCA (Montreal Cognitive Assessment), BL/OTH, ∆SO, t_0_, t_1_, t_2_ Apathy Scale, BL/OTH, ∆SO, t_0_, t_1_, t_2_ Covi Anxiety Scale, BL/OTH, ∆SO, t_0_, t_1_, t_2_ GSE (General Self-Efficacy Scale), BL/OTH, ∆SO, t_0_, t_1_, t_2_ MDS-UPDRS-III, BL/OTH, ∆SO, t_0_, t_1_, t_2_ SCOPA-sleep (Scales for Outcomes in PD – Sleep), BL/OTH, ∆SO, t_0_, t_1_, t_2_ marker of neuroprotection (BDNF, TNF-alpha, IL-6), BL/OTH, ∆O, t_0_, t_1_, t_2_ *descriptive* custom survey (satisfaction, usefulness, comprehensiveness, perceived burden, relevance of assigned intervention, timing, length, number of sessions) *evaluation timepoints:* t_0_ = baseline, t_1_ = after 12 wks, t_2_ = after 24 wks	*baseline* (↑) longer duration, higher doses, more extensive medical comorbidity (EXCEED) ↓↑ differences between the groups ↓ education, L-Dopa-dosage (SGE) *combined group effects* ↑ SCOPA-sleep (24 wks) ↑ MoCA (24 wks) ↑ BDNF (12 wks, 24 wks) ↓↑ Apathy scale, Covi Anxiety Scale, GSE, MDS-UPDRS-III ↓ MADRS (12 wks, 24 wks) descriptive * participation in program is useful (100% EXEED, 84.6% SGE) satisfaction with social aspects of group attendance (EXCCED) easy to fit exercise into their lives (SGE) fixed-time groups were difficult (EXCEED) *evidence level* II
	SGE (self-guided CDSM program + exercise)						12 wks SGE self-guided, same CDSM information like EXEED (written material) participants read and practice it on their own single initial in-patient group orientation exercise program exercises (3 per wk) with written instructions phone calls to self-report (1 per wk, first 12 wks)		
Hellqvist et al., 2020 Sweden	NPS (National Parkinson School)	(1) outcomes of the NPS from the perspective of the participants using self- reported questionnaires	case-control study, quasi-experimental clinical practice, monocenter, additional formative evaluation *intervention group* PwPD (*n* = 70) CG (*n* = 41) *control group* PwPD (*n* = 62) CG (*n* = 34) *comments:* age and gender matched control group, power >0.80 (PDQ-8), twice sample size	need of disease related knowledge to understand how it affect the daily life, stress management, communication, anxiety and depression, self-monitoring, enriching activities, future life with PD	self-management and self-monitoring as central concepts, knowledge and tools to enhance ability to live and handle life with disease, awareness about thoughts and reactions, replace negative thoughts with constructive thoughts helps manage difficulties	relaxation exercises (15 min, end of a session)	*intervention group* 7 wk NPS interactive group sessions (1 per wk, 2 h) introduction of a specific topic to give more knowledge, group discussion, practical exercises, relaxation exercises and homework qualified trainers (health care professionals) CG: common session with PT *control group* standard care	HandY, BL/OTH, t_0_ PADLS (PD Activities of daily living scale), BL/OTH, t_0_ (PT) sociodemographic data, BL/OTH, t_0_ PDQ-8 (Parkinson’s Disease Questionnaire-8) (PT) Euroqol-5D (PT, CG) ZBI (Zarit Burden Index) (CG) LitSat-11 (Life satisfaction Checklist), (PT, CG) PSF-16 (Parkinson Fatigue Scale) (PT) item 1 of RAND-36-questionnaire (PT, CG) heiQ (Health Education Impact Questionnaire) for program evaluation (PT, CG) *evaluation timepoints:* t_0_ = baseline, t_1_ = after 7 wk	*baseline* ↑ male participants (intervention group) ↓↑ difference between groups *PT (intervention group)* ↑ EuroQol-5D ↑ heiQ subscales (“constructive attitudes and approaches”, “skill and technique acquisition”) ↓ PDQ-8 *PT (control group)* ↓ LitSat-11 subscales (“satisfaction with life as a whole”, “leisure”, “contacts”) *CG* (↑) improvement of all scores after program ↓↑ difference between groups ↓ LiSat-11 subscale (“satisfaction with life as a whole”) *heiQ* ↑ relevant content, understanding of PD (↑) CG find NPS more helpful than PT in terms of goal setting self-reported confounding factors * (health problems, deaths in family, birth grandchildren) *evidence level* III
Lindskov et al., 2007 Sweden	multi-disciplinary group educational program with caregiver	(1) evaluate patient-reported health outcomes of a multi-disciplinary group educational program as part of routine clinical practice	naturalistic non-randomized controlled trial, monocenter waiting list *intervention group* PwPD (*n* = 49) *control group* PwPD (*n* = 48) with CG *comments:* power > 0.80 (standard error, SF-12)	general information (e.g., symptoms, disease progression), medical and surgical treatment, nutrition, oral hygiene, availability of funds, applying for funds, social support	managing day-to-day disease-related problems, focusing on possibilities rather than limitations, coping strategies	relaxation, speech and movement exercises	*intervention group* 6 wk multidisciplinary group educational program group sessions (1 per wk, 2 h, 6–8 participants) lecture, interactive discussion, exercises multidisciplinary team (nurse, physician, occupational therapist, dietician, psychologist, speech therapist, dental hygienist, social worker) CG: 1st, 2nd hour separate *control group* delayed intervention after follow-up	HandY, BL/OTH, t_0_ sociodemographic data, BL/OTH, t_0_ SF-12 (12 item short-form health survey), BL/OTH, PO, t_0_, t_1_ *evaluation timepoints:* t_0_ = baseline, t_1_ = after 10 wk	*baseline* ↓↑ difference between groups *post intervention* ↑ L-Dopa-dose (control group) ↓↑ SF-12 *evidence level* III
Lyons et al., 2020 USA	Strive to thrive: Self-Management for Parkinson’s Disease	(1) exploration of health benefits, self-management behaviors, illness communication for couples participating together in an existing community-based self-management workshop for PD	case-control study, quasi-experimental’, multicenter, waiting-list design *intervention group* PwPD + CG (couples, *n* = 19) *control group* PwPD + CG (couples, *n* = 20)	PD-specific content not further described, depression, sleep problems	self-management skills like monitoring, taking action, problem-solving, decision-making and evaluating results	exercises (not further described), relaxation techniques	*intervention group* 7 wk Strive to Thrive 6 wk according to CDSMP (Chronic Disease Self-Management Program) adding 1 wk with pd-specific content group intervention peer trainees trained by co-principal investigator (master trainer, 4 day training session Stanford University) Stanford Self-Management Program Fidelity Manual *control group* wait list/delayed intervention	sociodemographic data, BL/OTH, t_0_ (PT, CG) SF-36 (36 item short-form health survey), ∆PO, t_0_, t_1_ (PT, CG) CES-D (Center for Epidemiologic Studies-Depression scale), ∆PO, t_0_, t_1,_ (PT, CG) MCSI (multidimensional Caregiver Strain Index), ∆PO, t_0_, t_1_ (CG) evaluation questionnaire CDSMP-Curriculum (self-management, self-efficacy), ∆PO, t_0_, t_1,_ (PT, CG) active engagement und protective buffering (VAS Scale), ∆PO, t_0_, t_1,_ (PT, CG) *evaluation timepoints:* t_0_ = baseline, t_1_ = after 7 wk	*baseline* ↓↑ differences between groups ↓ aerobic activity, physical health (intervention group (PT)) *PT* (↑) aerobic activity (↑) mental relaxation (↑) self-management behaviors (↓) physical health (↓) engage in less protective buffering (↓) self-efficacy to manage PD *CG* ↑ improvement in engagement in mental relaxation techniques (↑) care strain (↑) engagement in strength-based activities (↑) self-efficacy to support partners in managing PD ↓↑ physical health ↓↑ aerobic activity (↑) self-management behaviors (↓) depressive symptoms (↓) engage in less protective buffering *evidence level* III
Gruber et al., 2008 Canada	EMP (The Early Management Program)	differences between 2 locations: (1) program evaluation (2) participants characteristics (3) attendance and non-completion rates (4) immediate benefits in terms of self-reported and physical outcomes	pre/post-test design, summative evaluation, 2 centers study *Baycrest group* PwPD (*n* = 40) HandY = 1–2 < 3 y disease duration location: Toronto *CMID group* PwPD (*n* = 52) HandY = 1–2 < 3 y disease duration location: Markham no CG	medication, pain, sleep, being an informed healthcare consumer, relationships (loving and caring), mind, emotions and behavior, participation in aerobic activities	programs based on self-management approach, aim to optimize ability to live well with PD, personal goal setting, coping with change and PD	*Axial Mobility Program:* exercises for flexibility, strength, posture, balance, relaxation techniques, walking, speech and swallowing	8 wk EMP group intervention (1 per 2 wk, 2 h) 1st hour interactive discussions 2nd hour exercises short-term goals (every 2 wk) long term goal (completion by end of program) provided by a physiotherapist and a trained volunteer facilitator	*(PO not defined)* UPDRS part I, III, BL/OTH, t_0_ HandY, BL/OTH, t_0_ AI (Activity Inventory of the Chedoke McMaster), BL/OTH, t_0_ BBS (Berg Balance Scale), BL/OTH, t_0_ sociodemographic data, BL/OTH, t_0_ CISM (chronical illness self-management questionnaire), ∆O, t_0_, t_1_ FR (functional reach), ∆O, t_0_, t_1_ timed functional movements, walking speed, ∆O, t_0_, t_1_ FAR (functional axial rotation), ∆O, t_0_, t_1_ *evaluation timepoints:* t_0_ = 2 wks prior to beginning of EMP, t_1_ = after 8 wk (last session)	*baseline* ↑ age (CMID) ↑ month since diagnosis (CMID) ↑ UPDRS part I (CMID) *post intervention* ↑ CISM subscales (stretching, cognitive symptom management, mental stress management communication with physician) ↑ FAR (only Baycrest) ↑ FR ↑ timed functional movements, walking speed (↑) CISM aerobic subscale *evidence level* IV
Horne et al., 2019 Australia	Parkinson’s disease Wellbeing Program	(1) short-term improvements in psychosocial and physical parameters and sustainability at 12-mth follow-up (2) influence of older patient age, lower MMSE, higher HandY stage and disease duration on baseline parameters and physical improvement at 12 months (3) association of baseline patient characteristics and history of falls (4) relationship between baseline characteristics, exercises, 12-mth balance and psychosocial parameters	prospective observational study, single center PwPD (*n* = 135), HandY 1–3 no CG	importance of exercise, nutrition and medication, communication, speech and swallowing, sleep and fatigue, falls, freezing and posture, stress management and independent living	motivation to exercise daily, not explicit mentioned	dual tasking, extension, rotation, reaching, stepping, symmetrical gait, cardiovascular warm-up, stretching	5 wk Wellbeing Program group sessions (2 per wk, 2.5 h, 6 participants) education (1 h) exercises (1 h 10 min), adapted to individual needs and preferences general discussion (20 min) clinic physiotherapist, exercises physiotherapist handouts home exercise program with written explanations and daily exercise diary exercise guidelines	(*PO not defined*) MMSE, BL/OTH, t_0_ HandY, BL/OTH, t_0_ sociodemographic data, BL/OTH, t_0_ *physical measures* fast gait velocity over 10 m, O, t_0_, t_1_, t_2_ 2 MW (distance walked in 2 min), O, t_0_, t_1_, t_2_ TUG, (timed up and go), O, t_0_, t_1_, t_2_ STS (number of Sit to stand in 30 s), O, t_0_, t_1_, t_2_ BBS (Berg Balance Score), O, t_0_, t_1_, t_2_ *psychosocial measures* PDQ-39, O, t_0_, t_1_, t_2_ DASS-21 (Depression Anxiety Stress Score), O, t_0_, t_1_, t_2_ PSF-16, O, t_0_, t_1_, t_2_ *evaluation timepoints*: t_0_ = baseline, t_1_ = after intervention (6 wk), t_2_ = 12-month-follow-up (17 wk)	*after 6 wks* ↑ physical measures (2 MW, STS, TUG, gait velocity and BBS) ↑ DASS-21 ↓ PDQ-39 ↓ PFS-16 *after 12 mths* ↑ physical measures (2 MW, STS, TUG, gait velocity and BBS) ↓↑ DASS-21 ↓↑ PDQ-39 ↓↑ PFS-16 *regression analysis* worse physical parameters at baseline associated with older age, lower MMSE, higher HandY worse psychosocial parameters at baseline associated with lower MMSE, higher HandY improvement in physical parameters (12 wk) predicted by MMSE, HandY, PFS-16, patient age *evidence level* IV
Sunvisson et al., 2001 Sweden	Multi-disciplinary group educational program	(1) Evaluation of a training program for PwPD (2) influence on psychosocial situation, ability to handle daily life activities and mobility pattern	single group design, monocenter pre/post-test design PwPD (*n* = 45) HandY ≤ 4 no CG	physical/psychological symptoms, dialectical liaison between body and mind, medical treatments and side-effects, influences from physical surroundings and social networks	based on structure of connection model (interaction between person and environment), manage sickness-related difficulties in daily life by exploring limitations and possibilities, how to obtain and maintain good self-care	coordination, balance, body rhythm, stretching, relaxation and body language, practical advice: rise from chair, turn around in and get out of bed	5 wk multidisciplinary group education program interactive group sessions (2 per wk, 2 h) 1 h dialogue and 1 h physical exercises provided by nurse and physiotherapist tasks at home, handout	PLM (postural-locomotor-manual), BL/OTH, ∆PO, t_0_, t_1_, t_2_ HandY, BL/OTH, ∆SO, t_0_, t_1_, t_2_ UPDRS (ADL, motor examination), BL/OTH, ∆SO, t_0_, t_1_, t_2_ SIP (sickness impact profile), BL/OTH, ∆SO, t_0_, t_2_ *evaluation timepoints:* t_0_ = baseline, t_1_ = after intervention (5 wk), t_2_ = 3-month-follow-up (17 wks)	↑ PLM subscales movement time, simultaneous index/level of integrated movements ↑ improvement SIP and SIP subscales psychosocial dysfunction, sleep and rest (baseline + 17 wk) ↓↑ UPDRS subscale motor examination ↓ UPDRS subscale ADL (baseline+ 5 wks, 5 wks + 17 wks) *evidence level* IV
Chaplin et al., 2012 UK	Hertfordshire Neurological Services Self-Management Program	(1) description of program development (2) discussion of implications for service providers and future research	program development and concept process evaluation persons with long-term neurological conditions (*n* = 60) CG na	symptoms, medication, psychological aspects, communication, nutrition, advice for speech and swallowing difficulties, strategies or enhancing function and mobility-circuits	based on main theoretical approaches to self-management (social cognitive theory and self-regulation model), personal health plans, self-management concept and support tools, strategies for daily life and coping	exercise examples and physiotherapy	condition-specific self-management groups at Hertfordshire neurological service 3 modules (self-management, living well, disease-specific (PD)) group discussion, handouts multidisciplinary team (nurse, psychologists, physiotherapists, occupational therapists, dieticians, rehabilitation assistants) number of wk and participants, length of sessions not further described CG: only first module combined with PT	evaluation questionnaire, t_1_ *evaluation timepoint:* t_1_ = after intervention	most helpful outcome: discussions/other people’s experiences (>50%) high level of satisfaction need of inclusion of CG (25%) *evidence level* V
van Nimwegen et al., 2010/2013 Netherlands ^#^	ParkFit Program	(1) development of a multifaceted intervention to promote physical activity in sedentary PwPD (2) investigation whether this program affords increased physical activity levels that persist for two years (3) search for possible health benefits and risks of increased physical activity	RCT, multicentre *intervention group* PwPD (*n* = 299) HandY ≤ 3 *control group* PwPD (*n* = 287) HandY ≤ 3 no CG *comments:* 32 participating hospitals, Power 0.80	general information about PD benefits of physical activity behavioural change strategies like identifying and overcoming any perceived barriers to engage in physical activity	combination of techniques based on models of behavioural change identify individual beliefs goal setting, recruiting social support	physical therapy	*intervention group* 2 y ParkFit max. 19 (1st year)/23 (2nd year) physical therapy sessions a year (30 min) max 16 (1st year)/12 (2nd year) coaching sessions a year experienced trained physical therapists of Dutch ParkinsonNet (attention to techniques of behavioural change strategies) brochure with specific behavioural change strategies workbook with health contract (physiotherapist and PT) logbook (monitoring of 6-month-goals) activity monitor with visual feedback (triaxial accelerometer) personalized website shows the activity history	education, employment, lifetime physical activity, BL/OTH, t_0_ attitude, social support, self-efficacy towards physical activity, BL/OTH, t_0_ (only ParkFit) blood pressure, height, body weight, BL/OTH, t_0_, t_4_, t_6_ alcohol use, smoking, BL/OTH, t_0_, t_4_, t_6_ LAPAQ (LASA physical activity questionnaire), PO, t_0_, t_3_, t_4_, t_5_, t_6_ 6 MWT (six minute walk test), SO, t_0_, t_4_, t_6_ level of physical activity (time, kilocalories), SO, t_1_ PDQ-39, SO, t_0_, t_3_, t_4_, t_5_, t_6_ UPDRS III, O, t_0_, t_4_, t_6_ Nine hole peg board test, O, t_0_, t_4_, t_6_ TUG, O, t_0_, t_4_, t_6_ SCOPA-sleep, O, t_0_, t_3_, t_4_, t_5_, t_6_ HAD-S, O, t_0_, t_3_, t_4_, t_5_, t_6_ FSS (Fatigue Severity Scale), O, t_0_, t_3_, t_4_, t_5_, t_6_ cognitive functioning tests, O, t_0_, t_4_, t_6_ Åstrand-Ryhming test, O, t_0_, t_2_, t_4_ DXA (dual energy X-ray absorptiometry), O, t_0_, (subgroup of 300 PT) PD medication, O, t_0_, t_3_, t_4_, t_5_, t_6_ medical costs and EuroQol-5D, O, t_0_, t_3_, t_4_, t_5_, t_6_ number of falls, O, t_0_, t_2_, t_3_, t_4_, t_5_, t_6_ *evaluation timepoints:* t_0_ = baseline, t_1_ = per week, t_2_ = monthly, t_3_ = after 6 mths, t_4_ = after 12 mths, t_5_ = after 18 mths, t_6_ = after 24 mths	*6 to 24 mth (change)* ↑ level of physical activity ↓↑ LAPAQ ↓↑ PDQ-39 ↓↑ number of falls (↓) 6 MWT *evidence level* I
	ParkSafe Program			general information about PD aims and benefits of physical therapy importance of safety on daily activities	not included	interventions from physical therapy guidelines for PD to move more safely improving quality of transfers	*control group* 2 y ParkSafe max. 35 sessions a year (30 min) individualized physical therapy program experienced physical therapists of Dutch ParkinsonNet brochure (benefits of physical therapy) bi-annual newsletter		

Table shows self-management education (SME) programs with quantitative evaluation studies. Changes in outcomes are indicated as follows: ↑ significant increase, ↓ significant decrease, (↑) trending increase, (↓) trending decrease, ↓↑ no change, na not applicable. * summarized for space restrictions, ^#^ This study was not identified by the search terms of the current systematic review, because of the usage of the term “behavioral program” instead of “self-management”. Despite this, the study was still reported because of its relevance to the overarching theme of the review. Abbreviations: AACPDM American Academy for Cerebral Palsy and Developmental Medicine, RCT randomized controlled trial, MKP multimodal complex treatment, QOL quality of life, HRQOL health-related quality of life, PwPD patients with Parkinson’s disease, PD Parkinson’s disease, *n* number, y years, wk week(s), mth month, h hours, min minutes, s seconds, t time, PO primary outcome, SO secondary outcome, PL/OTH baseline/others, PT patients, CG caregiver, PEEP Patient Education Program for PD, HandY Hoehn and Yahr Scale, MMSE Mini Mental State Examination, ADL Activities of Daily Living Scale, VAS visual analogue scale, SDS Self-rating Depression Scale, BELA-P-k Belastungsfragebogen Parkinson Kurzversion, BELA-A-k Belastungsfragebogen Parkinson Angehörige Kurzversion, UPDRS Unified Parkinson’s Disease Rating Scale, PDQ-39 Parkinson’s Disease Questionnaire-39, PDQ-SI Parkinson’s Disease Questionnaire-Summary Index, FKV-LIS-SE Freiburg Coping with Disease Questionnaire, SOC-29 Sense of Coherence Scale, GSE General Self-Efficacy Scale, HAS-D German Hospital Anxiety and Depression Scale, GDS Geriatric Depression Scale, SEADL Schwab and England ADL scale, SDS Zung Self-Rating Depression Scale, PMS Global patient’s mood status, CMS Caregiver mood status, EXCEED exercise therapy for PD, CDSM chronic disease self-management, CCI Charlson Comorbidity Index, MARDS Montgomery-Asberg Depression Rating Scale, MoCa Montreal Cognitive Assessment, GSE General Self-Efficacy Scale, SCOPA-sleep Scales for Outcomes in PD–Sleep, CMID Centre for Movement Disorders in Markham, SGE self-guided CDSM program + exercise, SF-12 12 item short-form health survey, EMP The Early Management Program, AI Activity Inventory of the Chedoke McMaster, BBS Berg Balance Scale, CISM chronical illness self-management questionnaire, FR functional reach, FAR functional axial rotation, PADLS PD Activities of daily living scale, PDQ-8 Parkinson’s Disease Questionnaire-8, ZBI Zarit Burden Index, LitSat-11 Life satisfaction Checklist, PSF-16 Parkinson Fatigue Scale, heiQ Health Education Impact Questionnaire, NPS National Parkinson School, SF-36 36 item short-form health survey, CES-D Center for Epidemiologic Studies-Depression scale, MCSI multidimensional Caregiver Strain Index, 2 MW distance walked in 2 min, TUG timed up and go, STS number of Sit to stand in 30 s, BBS Berg Balance Score, DASS-21 Depression Anxiety Stress Score, SIP sickness impact profile, PLM postural-locomotor-manual, LAPAQ LASA physical activity questionnaire, 6 MWT six-minute walk test, FSS Fatigue Severity Scale, DXA dual energy X-ray absorptiometry).

**Table 2:** Qualitative evaluation studies.

Author, Date, Country	Program	Study Goals	Study Design/ Population	Intervention Content			Intervention Format	Measurement Instruments, Evaluation Timepoints	Outcome
				Information Provision	Behavioral Modification	Physical Exercises			
Hellqvist et al., 2018 Sweden	NPS (National Parkinson School)	(1) identify experiences valuable for managing daily life after participation in the program (2) explore applicability of self- and family-management framework by Grey	qualitative explorative design with two-step-analyses, multicenter PwPD (*n* = 25) CG (*n* = 17)	need of disease related knowledge to understand how it affect the daily life, stress management, communication, anxiety and depression, self-monitoring, enriching activities, future life with PD	self-management and self-monitoring as central concepts, knowledge and tools to enhance ability to live and handle life with disease, awareness about thoughts and reactions, replace negative thoughts with constructive thoughts helps manage difficulties	relaxation exercises (15 min, end of a session)	7 wk NPS interactive group sessions (1 per wk, 2 h) introduction of a specific topic to give more knowledge, group discussion, practical exercises, relaxation exercises and homework qualified trainers (health care professionals) CG: common session with PT	demographic questionnaire, t_0_ 5 audio-record and verbatim transcription of last intervention session (reflection by participants on NPS, expected use of knowledge, technique in daily lives), t_1_ inductive thematic analyses of transcript deductive application to self- and family-management framework by Grey of transcript *evaluation timepoints:* t_0_ = onset of NPS t_1_ = wk 7, last intervention session	*major themes* of being an NPS participant exchanging experiences and feeling support adjustment and acceptance of PD for managing daily life promoting life satisfaction * self- and family-management framework fit to persons with PD and their relatives
Hellqvist et al., 2020 Sweden		(1) whether PwPD and CG implemented the strategies of self-monitoring included in the NPS and use them in clinical encounters with health care professionals	qualitative inductive study with two-part method: observation and follow-up interviews, monocenter PwPD (*n* = 10) CG (*n* = 3)					observation during a routine visit at outpatient clinic (45 to 60 min) with observational guide (relational interaction, social processes, content of topics discussed during the consultation), t_1_ interview without physician after consultation with interview guide (3 to 22 min), t_1_ *evaluation timepoints:* t_1_ = 3 to 5 mth after participation on NPS intervention	NPS have an impact on understanding PD and abilities available to handle everyday life PT and CG use techniques of self-observation in everyday lives * *core category* awareness of own abilities strengthens mutual understanding and communication in the health care encounter *subcategories* self-observation in everyday life self-care activities to promote health managing the emotional impact of PD
Mulligan et al., 2011 New Zealand	Living Well with Parkinson’s Disease	(1) evaluate an innovative self-management program from the users’ perspectives	qualitative evaluation study, individual interviews with participants, monocenter PwPD (*n* = 8) CG (*n* = 3)	knowledge about PD current research, medication, nutrition, emotional and psychological aspects	enable participants to effectively self-manage life, identify level of self-efficacy	physical exercises (not further described)	6 wk Living Well with PD group intervention (90 min) lecture and interactive discussion morning or evening sessions at beginning of program: PDQ-39 (Parkinson’s Disease Questionnaire-39) and CDSES (Chronic disease self-efficacy scale) to highlight personal perceived level of self- efficacy in relation to living with the symptoms different health care providers (dietician, not further described) CG: common session with PT	semi-structured individual interviews (10 to 30 min) interview topics: participant’s background of PD, expectations of the program, perceived outcomes of the program, organizational aspects of the six seminars audio-record and verbatim transcription general inductive analyses *evaluation timepoint:* t_1_ = 2 to 7 wk. after participation on intervention	strong need for knowledge of living with PD program improved ability to cope and to self-manage living with PD learning new information, meeting other people with PD reported psychosocial benefits participants’ recommendations for future follow-up sessions * *core categories* the before: me and my Parkinson’s, reasons for attending, knowledge the after: psychosocial benefits, self-management/social benefit, new strategies, reinforcement, valuable content, logistics that enhanced the future: content, logistics that detracted, philosophy of self-management

Table shows studies using qualitative methods to evaluate self-management and patient programs. * summarized for space restrictions. Abbreviations: NPS National Parkinson School, PwPD patients with Parkinson’s disease, PD Parkinson’s disease, *n* number, wk week(s), mth month, h hours, min minutes, t time, PT patients, CG caregiver, PDQ-39 Parkinson’s Disease Questionnaire-39, CDSES Chronic disease self-efficacy scale.

**Table 3:** Evaluation concepts for future studies.

**Author, Date, Country**	**Program**	**Study Goals**	**Study Design** **Population**	**Intervention Content**			**Intervention Format**	**Measurement Instruments** (BL/OTH (baseline, others (e.g., confounder)), PO (primary outcome), SO (secondary outcome), O (outcome not defined as PO or SO))) **Evaluation Timepoints**	**Intended Outcome**
				**Information Provision**	**Behavioral Modification**	**Physical Exercises**			
Siegert et al., 2019 Germany	ParkPro- Train	(1) user-centered development and implementation of an individualized tablet-based training program (2) transfer of the physically activating exercises learned in the MKP and other physical activities into everyday life (3) improvement in QOL, social participation and delayed progression of impairment through regular implementation of the program	*mixed methods* (1) monocenter, quasi-randomized longitudinal study, RCT (2) interviews and focus groups (3) formative evaluation (tablet-based program, administration panel) (4) evaluation of the training program implementation *intervention group* PwPD (*n* = 133) *control group* PwPD (*n* = 133) no CG *comments:* calculation based on PDQ-8 (power >0.80)	knowledge, preparing for everyday life at home	based on HAPA-model (intent formation and implementation of health behavior), based on 5-A-model (increase self-management skills and support behavioral change), adaption of physical exercises (learning method of behavior shaping), considering of personal barriers and strategies to overcome those	different exercises promoting endurance, strength, balance and activities like Nordic walking, Tai Chi or dancing	*intervention group* during inpatient multimodal complex Parkinson therapy (MKP) tablet-app with physical activities (videos, instructions) and different degrees of difficulty or number of repetitions matching activities with patients’ abilities, needs and preferences planning individual home training program (with physiotherapist) for 3 wk with 3 30 min sessions per wk 2 group sessions at beginning *control group* usual inpatient multimodal complex Parkinson therapy (MKP)	*quantitative* sociodemographic data, BL/OTH, t_0_, t_3_ body height, weight, BL/OTH, t_0_, t_3_ use of health services, BL/OTH, t_0_, t_3_ PDQ–8 (Parkinson’s Disease Questionnaire-8), PO, t_0_, t_1_, t_3_ IMET (Index Messung von Einschränkungen der Teilhabe), SO, t_0_, t_3_ FES-I (Falls Efficacy Scale International Version), SO, t_0_, t_1_, t_3_ PDSS-2 (Parkinson’s disease Sleep Scale), SO, t_0_, t_1_, t_3_ PHQ-4 (Health Questionnaire for Patients), SO, t_0_, t_1_, t_3_ SCQ-D (Comorbidity Questionnaire of Sangha), SO, t_0_, t_3_ Federal Health Survey 1999 (physical activity), SO, t_0_, t_3_ pain (single item 5-point-scale), SO, t_1_ performance capability (numerical scale 0 to 10), SO, t_0_, t_3_ *qualitative* interviews with 16 PT t_2_, t_3_ (feasibility of implementing the training plan at home, wishes and needs interviews with physiotherapists t_1_ focus group with health care professionals (practicality of the app, benefit for therapeutic and medical work during MKP and preparation for the time at home) *evaluation timepoints:* t_0_ = baseline/right before MKP, t_1_ = 3 wk. follow-up/right after MKP, t_2_ = 9 wk after MKP, t_3_ = 9 mth after t_1_	↑ physical activity ↑ improvement of motor and non-motor impairments ↑ QOL ↑ long time effects of MKP ↓ individualized, time-consuming care by a therapist ↓ costs for conventional occupational/physiotherapy prescriptions *descriptive* acceptance and adherence
Navarta-Sánchez et al., 2008 Spain	ReNACE	(1) improvement of QOL of PwPD and their family carers by means of a multidisciplinary psychoeducational intervention focusing on fostering coping strategies and their psychosocial adjustment to PD (2) evaluate the perceptions, opinions and satisfaction of the patients and family carers and explore the reflections of the social and healthcare providers involved in this intervention	*mixed methods* part of ReNACE research program (1) quasi-experimental study with control group (2) focus groups PwPD (*n* = 104) CG (*n* = 106) *comments:* calculation based on PDQ-39 and SQLC	*ReNACE* getting to know PD, healthy life habits, resources, management of stress and complicated situations, look for information, normalize the situation and partake in activities, positive self-esteem, empathy and patience	*ReNACE* adapting and coping with PD and stressful situations, empowerment, awareness of participants cognitive and behavioral efforts	not contained in both interventions	*intervention group* 9 wk ReNACE group intervention (1 per wk, 90 min, 15–20 participants) multidisciplinary team (general practitioner, neurologist, social worker, psychologist, primary care nurse, expert patient) standardized content and methodology manual CG: same topics in separate groups	*quantitative* sociodemographic data, others, t_0_ PDQ-39 (Parkinson’s Disease Questionnaire-39), PO, others t_0_, t_1_, t_2,_ (PT) SQLC (Scale of Quality of Life of Caregivers), PO, t_0_, t_1_, t_2,_ (CG) PAIS-SR (Psychosocial Adjustment to Illness scale), SO, t_0_, t_1_, t_2_ (PT, CG) Brief COPE scale, SO, t_0_, t_1_, t_2_ (PT, CG) *qualitative* focus groups with PTs, CGs, t_2_ (intervention group) (benefits in terms of coping skills and psychosocial adjustment and QOL, opinion on issues and methodology) focus groups with HCP (future implementation, time needed and costs, integration as part of care pathways) *evaluation timepoints:* t_0_ = baseline, t_1_ = after intervention, t_2_ = 6 mth after t_1_	↑ psychosocial adjustment to PD ↑ QOL of PwPD and CG ↑ compliance with drug treatment and healthy lifestyles
	GEP (general education program)			*GEP* general information on PD, healthy life habits, resources in the community	*GEP* not mentioned		*control group* 5 wk GEP group intervention (1 per wk, 90 min, 15–20 participants) social and health care professionals CG: same topics in separately sessions		
King et al., 2015 USA	ABC-C (Agility Boot Camp-Cognition)	(1) improvement of mobility and/or cognition after partaking in the ABC-C program compared to a control intervention (2) prediction of cognition and postural, cognitive and brain posture/locomotor circuitry deficits for responsiveness to the cognitively challenging ABC-C program	cross-over RCT PwPD (*n* = 120) age 50–90 y no CG *comments:* power calculation based on Mini-BESTest	*ABC-C* not mentioned	*ABC-C* not included	*ABC-C* gait training, lunging, PWR! moves, agility, boxing, Thai Chi	*intervention group* 6 wk ABC-C training circuit as group intervention (3 per wk, 80 min, 6 participants) different levels of difficulty, sequences, supports, with cognitive tasks certified exercise trainer	*quantitative:* Mini-BESTest (Mini Balance-Evaluation-System-Test), PO, t_0_, t_1_, t_2_ MDS-UPDRS (Movement Disorders Society Unified Parkinson’s disease rating scale), SO, t_0_ NFOGQ (new freezing of gait questionnaire), SO, t_0_ ABC (activities of balance confidence), SO, t_0_ PDQ-39, SO, t_0_ high angular resolution diffusion imaging (with tractography), SO, t_0_ rsfcMRI (communication between spatially disparate neural regions) SO, t_0_ balance (postural sway during 30 s of quiet stance with/without cognitive task), SO, t_0_, t_1_, t_2_ turning (1 min turning in place and turns during 2 min walk with/without a cognitive task), SO, t_0_, t_1_, t_2_ gait (spatial and temporal gait metrics during walking with/without dual task), SO, t_0_, t_1_, t_2_ SCOPA-COG (Scales for outcome of Parkinson’s disease-Cognition), SO, t_0_, t_1_, t_2_ Stroop task, flankers, Go/nogo, Stop signal task, SO, t_0_, t_1_, t_2_ Dot counting task, SO, t_0_, t_1_, t_2_ Benton judgment of line orientation test, SO, t_0_, t_1_, t_2_ *evaluation timepoints:* t_0_ = baseline, t_1_ = after 1st 6 wk intervention (before cross over), t_2_ = after 2nd 6 wk intervention	↓ executive function deficits and reduced structural and/or functional connectivity of the locomotor circuitry predict poor responses to challenging balance rehabilitation
	EPCD (education program for chronic disease)			*EPCD* finding information on PD, communicating effectively with health care providers, sleep, pain, fatigue, nutrition, medication, difficult emotions, stress, depression	*EPCD* not explicit mentioned, stress management and finding information	*EPCD* relaxation sessions, improving communication (verbal, voice tone, body language)	*control group* 6 wk EPCD group intervention (1 per wk, 90 min, 6 participants) relaxation sessions at home (5 per wk, 30 min)		
Gruber et al., 2008 Canada	SMandFPP (Safe Mobility and Falls Prevention Program)	(1) use feedback from participants to review and modify the program (2) assessment of adherence with HSEP (home support exercise program), fear of falling, improvement in fall risk factors, satisfaction with social participation	formative pilot evaluation (no results during publication), concept for outcome assessment PwPD (*n* not calculated) HandY = 3–4 no CG	prevention of falls, maximizing safe mobility through medications, exercise strategies, adaptive equipment	programs based on self-management approach, aim to optimize ability to live well with PD, goal setting and action plans	bed mobility transfers walking falls recovery transfers	7 wk SMandFPP group sessions (2 h) + booster session after 6 wk home support exercise program (HSEP)	*pilot evaluation* evaluation questionnaire focus groups *quantitative (PO not defined)* UPDRS part I, III, BL/OTH, t_0_, t_2_ number of falls, O, t_0_, t_1_, t_2_ CISM (Chronical Illness Self-Management Questionnaire), O, t_0_, t_1_, t_2_ adherence with HSEP (calendar), O, t_1_ BBS (Berg Balance Scale), O, t_0_, t_2_ TUG (Timed up and Go), O, t_0_, t_2_ ABC (Activities Specific Balance Confidence Scale), O, t_0_, t_2_ RNLI (reintegration to normal living Index, O, t_0_, t_2_ *evaluation timepoints:* t_0_ = baseline, 2 wk prior to beginning, t_1_ = at session 7, t_2_ = at session 8, after 6 wk	↑ improvement falls risk factors (BBS, TUG, ABC) ↑ satisfaction RNLI ↓ fear of falling (ABC) ↓ number of self-reported falls

Table shows study protocols for future studies on self-management programs. No related results have yet been published. Changes in outcomes (as hypothesized/postulated) are indicated as follows: ↑ significant increase, ↓significant decrease. Abbreviations: RCT randomized controlled trial, MKP multimodal complex treatment, QOL quality of life, PwPD patients with Parkinson’s disease, PD Parkinson’s disease, *n* number, y years, wk week(s), mth month, h hours, min minutes, s seconds, t time, PO primary outcome, SO secondary outcome, PT patients, CG caregiver, PDQ–8 Parkinson’s Disease Questionnaire-8, IMET Index Messung von Einschränkungen der Teilhabe, FES-I Falls Efficacy Scale International Version, PDSS-2 Parkinson’s disease Sleep Scale, PHQ-4 Health Questionnaire for Patients, SCQ-D Comorbidity Questionnaire of Sangha, GEP general education program, PDQ-39 Parkinson’s Disease Questionnaire-39, SQLC Scale of Quality of Life of Caregivers, PAIS-SR Psychosocial Adjustment to Illness scale, ABC-C Agility Boot Camp-Cognition, EPCD education program for chronic disease, Mini-BESTest mini balance-evaluation-system-test, MDS-UPDRS Movement Disorders Society Unified Parkinson’s disease Rating Scale, NFOGQ new freezing of gait questionnaire, ABC activities of balance confidence, SCOPA-COG Scales for outcome of Parkinson’s disease-Cognition.

**Table 4:** Required documentation and information about the program to be certified [67,68].

General Program Information	Formal Description	Proof of Effectiveness
Information about the applicant (institution, name etc.) Indication of target group/age group Duration of the measure Type of concept (original concept, license/franchise, adaptation); date of original version/version number Separate manual for trainers Separate manual for persons with chronic conditions Criteria for inclusion, exclusion, cancellation Detailed list of costs	Kind of conduct, place of conduct Caregiver-involvement Group size Group composition (closed vs. open etc.) Definition of learning objectives, contents, structure of the lessons etc. Relation between practical and theoretical units of the program Example of a lesson plan Methods Measures to avoid cancellation (e.g., motivational concept) Learning and teaching media Documentation Internal/external quality management and assurance Staff education (typically by a qualified interdisciplinary team including a specialized physician Train-the-Trainer workshops/certificates Required infrastructure (material and space resources)	Evaluation data If not yet available, detailed description of the evaluation concept Outcomes (proximal and distal)

## Supplementary Materials

The following are available online at https://www.mdpi.com/2077-0383/9/9/2787/s1, Figure S1: Study flow diagram, Table S1: PRISMA Checklist, Table S2: Other systematic reviews on different aspects of self-management in Parkinson’s disease.
