# Supplementary material for: Structured Care and Self-Management Education for Persons with Parkinson’s Disease: Why the First Does Not Go without the Second—Systematic Review, Experiences and Implementation Concepts from Sweden and Germany
Source: J Clin Med. 2020 Aug 28;9(9):2787. doi: 10.3390/jcm9092787 (PMC7563525; doi:10.3390/jcm9092787)
Supplement: Supplementary file 1 [file jcm-09-02787-s001.pdf]

Journal of
Clinical Medicine

MDPI

Supplementary Materials

Structured Care and Self-Management Education for Persons with Parkinson's Disease: Why the First does not Go without the Second—Systematic Review, Experiences and Implementation Concepts from Sweden and Germany

Jenny Tennigkeit [1,2], Tim Feige [1,1], Maria Haak [3], Carina Hellqvist [4], Ümran S. Seven [5], Elke Kalbe [5] Jaqueline Schwarz [6], Tobias Warnecke [7], Lars Tönges [8,9], Carsten Eggers [10] and Kai F. Loewenbrück [1,2*]

[1] Department of Neurology, University Hospital Dresden, 01307 Dresden, Germany; jenny.tennigkeit@uniklinikum-dresden.de (J.T.); tim.feige@uniklinikum-dresden.de (T.F.)
[2] German Center for Neurodegenerative Diseases (DZNE) Dresden, 01307 Dresden, Germany
[3] Faculty of Health Sciences, Kristianstad University, 291 88 Kristianstad, and Department of Health Sciences, Faculty of Medicine, Lund University, 221 00 Lund, Sweden; Maria.haak@hkr.se
[4] Department of Neurology, University Hospital Linköping, 58185 Linköping, Sweden; Carina.hellqvist@regionostergotland.se
[5] Medical Psychology, Neuropsychology and Gender Studies and Center for Neuropsychological Diagnostics and Intervention (CeNDI), Faculty of Medicine and University Hospital Cologne, University of Cologne, 50937 Cologne, Germany; Uemran.seven@uk-koeln.de (Ü.S.S.); elke.kalbe@uk-koeln.de (E.K.)
[6] Tumaini Institut für Präventionsmanagement GmbH, 01217 Dresden, Germany; jschwarz@tumaini.de
[7] Department of Neurology, University Hospital Münster, 48149 Münster, Germany; Tobias.Warnecke@ukmuenster.de
[8] Department of Neurology, St. Josef-Hospital, Ruhr-University Bochum, 44801 Bochum, Germany; Lars.toenges@rub.de
[9] Neurodegeneration Research, Centre for Protein Diagnostics (ProDi), Ruhr University, 44801 Bochum, Germany
[10] Department of Neurology, University Hospital Marburg, 35033 Marburg, Germany; Carsten.eggers@uk-gm.de
* Correspondence: Kai.loewenbrueck@uniklinikum-dresden.de; Tel.: +49-351-458-18518

Received: 31 July 2020; Accepted: 17 August 2020; Published: date

Supplementary Materials

Supplementary figures:

Figure S1: Study flow diagram

Supplementary tables:

Table S1: PRISMA Checklist

Table S2: Other systematic reviews on different aspects of self-management in Parkinson's Disease

Figure 1. Study flow diagram.

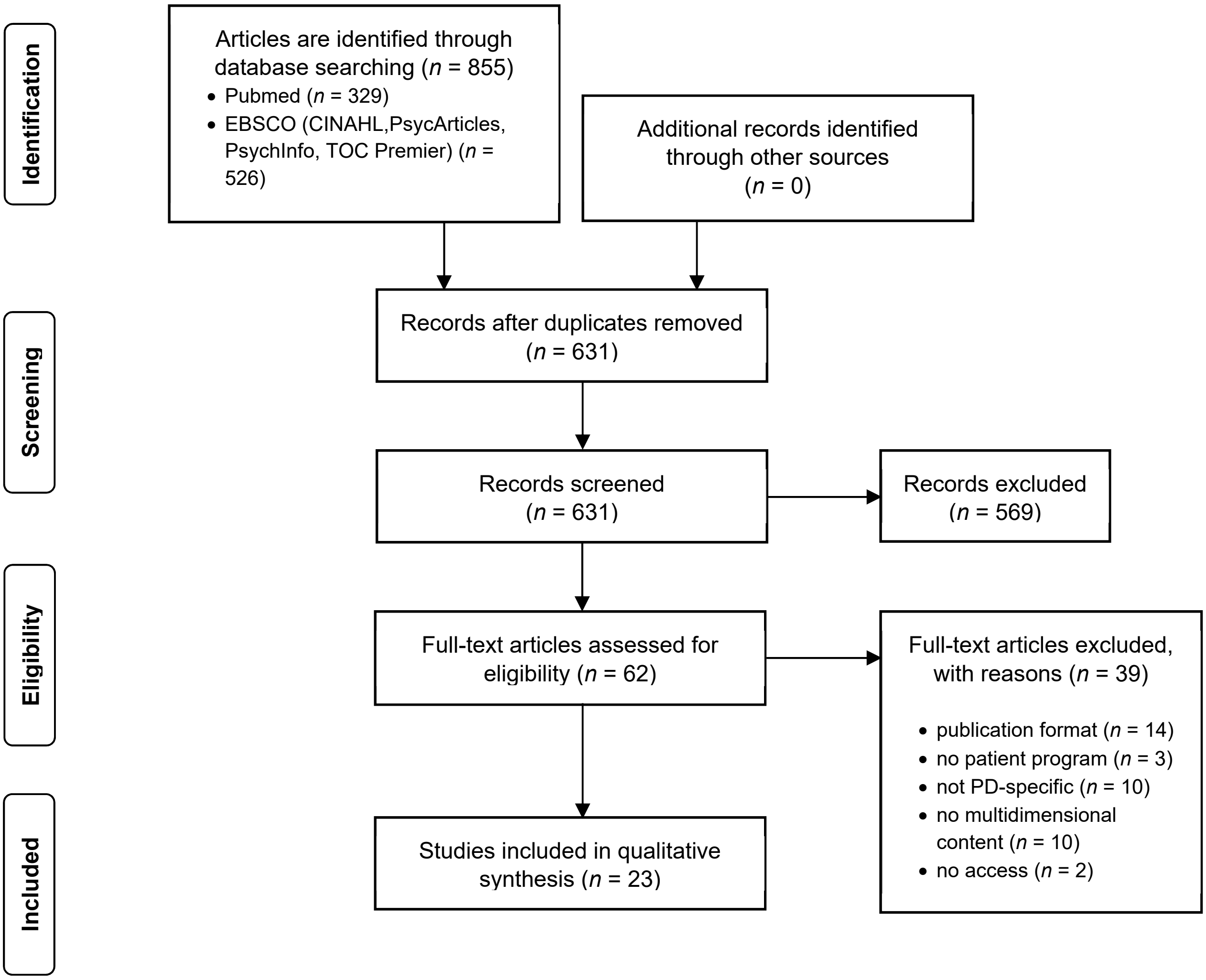

Study flow diagram according to PRISMA Statement [1] Abbreviations: n, number.

Table S1. PRISMA Checklist

**Section/topic**	#	**Checklist item**	**done**
Title	1	Identify the report as a systematic review, meta-analysis, or both.	✓
ABSTRACT			
Structured summary	2	Provide a structured summary including, as applicable: background; objectives; data sources; study eligibility criteria, participants, and interventions; study appraisal and synthesis methods; results; limitations; conclusions and implications of key findings; systematic review registration number.	✓
INTRODUCTION			
Rationale	3	Describe the rationale for the review in the context of what is already known.	✓
Objectives	4	Provide an explicit statement of questions being addressed with reference to participants, interventions, comparisons, outcomes, and study design (PICOS).	✓
METHODS			
Protocol and registration	5	Indicate if a review protocol exists, if and where it can be accessed (e.g., Web address), and, if available, provide registration information including registration number.	✓
Eligibility criteria	6	Specify study characteristics (e.g., PICOS, length of follow-up) and report characteristics (e.g., years considered, language, publication status) used as criteria for eligibility, giving rationale.	✓
Information sources	7	Describe all information sources (e.g., databases with dates of coverage, contact with study authors to identify additional studies) in the search and date last searched.	✓
Search	8	Present full electronic search strategy for at least one database, including any limits used, such that it could be repeated.	✓
Study selection	9	State the process for selecting studies (i.e., screening, eligibility, included in systematic review, and, if applicable, included in the meta-analysis).	✓
Data collection process	10	Describe method of data extraction from reports (e.g., piloted forms, independently, in duplicate) and any processes for obtaining and confirming data from investigators.	✓
Data items	11	List and define all variables for which data were sought (e.g., PICOS, funding sources) and any assumptions and simplifications made.	✓
Risk of bias in individual studies	12	Describe methods used for assessing risk of bias of individual studies (including specification of whether this was done at the study or outcome level), and how this information is to be used in any data synthesis.	
Summary measures	13	State the principal summary measures (e.g., risk ratio, difference in means).	
Synthesis of results	14	Describe the methods of handling data and combining results of studies, if done, including measures of consistency (e.g., I2) for each meta-analysis.	
Risk of bias across studies	15	Specify any assessment of risk of bias that may affect the cumulative evidence (e.g., publication bias, selective reporting within studies).	
Additional analyses	16	Describe methods of additional analyses (e.g., sensitivity or subgroup analyses, meta-regression), if done, indicating which were pre-specified.	✓
RESULTS			
Study selection	17	Give numbers of studies screened, assessed for eligibility, and included in the review, with reasons for exclusions at each stage, ideally with a flow diagram.	✓

Study characteristics	18	For each study, present characteristics for which data were extracted (e.g., study size, PICOS, follow-up period) and provide the citations.	✓
Risk of bias within studies	19	Present data on risk of bias of each study and, if available, any outcome level assessment (see item 12).	
Results of individual studies	20	For all outcomes considered (benefits or harms), present, for each study: (a) simple summary data for each intervention group (b) effect estimates and confidence intervals, ideally with a forest plot.	
Synthesis of results	21	Present results of each meta-analysis done, including confidence intervals and measures of consistency.	
Risk of bias across studies	22	Present results of any assessment of risk of bias across studies (see Item 15).	
Additional analysis	23	Give results of additional analyses, if done (e.g., sensitivity or subgroup analyses, meta-regression [see Item 16]).	
DISCUSSION			
Summary of evidence	24	Summarize the main findings including the strength of evidence for each main outcome; consider their relevance to key groups (e.g., healthcare providers, users, and policy makers).	✓
Limitations	25	Discuss limitations at study and outcome level (e.g., risk of bias), and at review-level (e.g., incomplete retrieval of identified research, reporting bias).	✓
Conclusions	26	Provide a general interpretation of the results in the context of other evidence, and implications for future research.	✓
FUNDING			
Funding	27	Describe sources of funding for the systematic review and other support (e.g., supply of data); role of funders for the systematic review.	✓

Table S2: Other systematic reviews on different aspects of self-management in Parkinson's Disease

Author, data, country	Title	Review goal	Search terms	Databases	Publications Retrieved (included in review)	Results / study designs	Overlap with current review (n, %)
Chandler et al., 2019<br>USA [2]	"Nonpharmacologic interventions for the self-management of anxiety in Parkinson's disease: a comprehensive review"	(1) identification of studies using self-management or components of self-managed nonpharmacologic interventions for the treatment of anxiety in individuals with PD	"Parkinson disease,", "Parkinson's disease," "Paralysis agitans,", "Anxiety," "Depression,", "Interventions," "Treatments," and "Self-management	Cochrane Database of Systematic Reviews, CINAHL, PubMed, PsycArticles	n=266<br>(n=13)	4 studies identified treatment of anxiety as primary goal (in other studies secondary goal)<br>In >50% of studies significant improvements common element: focused use of breath<br><br>5 RCTs, 8 quasi-experimental studies (uncontrolled)	1, 4,3%
Foster et al, 2014<br>USA [3]	"Systematic Review of the Effectiveness of Occupational Therapy–Related Interventions for People with Parkinson's Disease"	(1) description of documented effectiveness of interventions within the scope of occupational therapy practice<br><br>(2) suggestion of gaps in services, theory, and evidence for continued development.	Neurodegenerative, Parkinson, Parkinsonism, Parkinson's disease<br><br>Activities, activities of daily living, assistive devices, assistive equipment, caregiver support, cognition, cognitive behavioural therapy, community care, community programs, disease management, education, emotional regulation, exercise, extended care, fall prevention, health maintenance, health promotion, instrumental activities of daily living, intervention, leisure, lifts, mindfulness, mobility, mobility equipment, neurorehabilitation, nonmotor symptoms, occupational therapy, physical therapy,<br>physiotherapy, programs, quality of life, rehabilitation, rhythmic, scooters, self - management, services, sleep, social<br>engagement, therapy, treatment, walkers, wellness programs, wheelchairs, work, yoga	Medline,PsycINFO, CINAHL, AgeLine, OTseeker. Cochrane Database of Systematic Reviews, Campbell Collaboration	n= 4061<br>(n=55)	categories of interventions: exercise or physical activity, environmental cues, stimuli, and objects, self- management and cognitive–behavioural strategies<br><br>35 RCTs, 7 nonrandomized studies (cohort, case-control), 13 pre-post design, one group non-randomized	3, 13,0%
Hulbert et al. 2020<br>UK [4]	'Mind the gap' — a scoping review of long term, physical self-management in Parkinson's	(1) overview of apparent 'gap' between robust evidence base to promote long-term physical activity for PwPD and its provision, participation and uptake through physical self-management models	'Parkinson*' AND 'self-manag*'	Medline, EMBRASE, HMI, CDSR, Cochrane Methods Studies, DARE, CINAHL, PEDro, PsycINFO, Cochrane Library	n=1959<br>(n=19)	no consensus for the best model for physical self-management<br>combination of exercises and self-management<br>outcomes: impairments, activities, personal factors, QOL<br><br>4 RCTs, 3 reviews, 3 pre-post design, 6 cross-sectional design, 1 qualitative interview design, 2 mixed methods	3, 13,0%
Liddy et al. 2017<br>Canada [5]	Self-management support programs for persons with Parkinson's disease: An integrative review	(1) characteristics (structure and components) of self-management programs for PwPD<br><br>(2) evidence for the effectiveness of the self-management programs	Parkinson disease AND Self-manage* Self-care OR Self-help OR Self-directed OR Expert patient OR Health coach*	Medline, Embase, HealthSTAR, CINHAL, PsycINFO	n=82<br>(n=18)	89% of program specifically designed for PwPD<br>39% combined self-management support with other therapies<br>limited evidence to support specific self-management programs for PwPD<br><br>1 RCT, 1 review, 1 case control study, 3 pre-post evaluation studies, 1 post evaluation, 1 qualitative evaluation, 2 protocol descriptions	6, 26,1%
Owen et al. 2019<br>UK [6]	Falls Self-Management Interventions for People with Parkinson's Disease: A Systematic Review	(1) review of falls self-management interventions for PwPD<br><br>(2) assessment of efficacy for improving patient and caregiver outcomes, QOL and psychological outcomes.	free text terms to PD and self-management	MEDLINE, EMBASE, CINAHL, AMED, PSYCHInfo, Science Citation Index Expanded (Web of Science)	n=19242<br>(n=6)	each article evaluated the effect of self-management intervention alongside a physiotherapy intervention.<br>no intervention included caregiver<br>using group discussions and falls booklets<br>self-management often no main focus (incompletely described)<br><br>6 RCTs	1, 4,3%

Table S2 lists other systematic reviews found on self-management in Parkinson's Disease. In the column entitled "Overlap with current review", the number of studies are listed that were included both the respective listed former review and the current one.

PwPD patients with Parkinson's disease, PD Parkinson's Disease, RCT randomized controlled trial

References

1. Moher, D., A. Liberati, J. Tetzlaff, D.G. Altman, and P. Group, Preferred reporting items for systematic reviews and meta-analyses: the PRISMA statement. PLoS med, 2009. **6**(7): p. e1000097.

2. Chandler, S.K., J.L. Robins, and P.A. Kinser, Nonpharmacologic interventions for the self-management of anxiety in Parkinson's disease: A comprehensive review. Behavioural neurology, 2019. **2019**.

3. Foster, E.R., M. Bedekar, and L. Tickle-Degnen, Systematic review of the effectiveness of occupational therapy–related interventions for people with Parkinson's disease. American Journal of Occupational Therapy, 2014. **68**(1): p. 39-49.
4. Hulbert, S.M. and V.A. Goodwin, 'Mind the gap'—a scoping review of long term, physical, self-management in Parkinson's. Physiotherapy, 2020. **107**: p. 88-99.
5. Kessler, D. and C. Liddy, Self-management support programs for persons with Parkinson's disease: An integrative review. Patient education and counseling, 2017. **100**(10): p. 1787-1795.
6. Owen, C.L., K. Ibrahim, L. Dennison, and H.C. Roberts, Falls Self-Management Interventions for People with Parkinson's Disease: A Systematic Review. Journal of Parkinson's disease, 2019. **9**(2): p. 283-299.
